# Low-dose hypomethylating agents cooperate with ferroptosis inducers to enhance ferroptosis by regulating the DNA methylation-mediated MAGEA6-AMPK-SLC7A11-GPX4 signaling pathway in acute myeloid leukemia

**DOI:** 10.1186/s40164-024-00489-4

**Published:** 2024-02-20

**Authors:** Shuya Feng, Yigang Yuan, Zihan Lin, Min Li, Daijiao Ye, Liuzhi Shi, Danyang Li, Min Zhao, Chen Meng, Xiaofei He, Shanshan Wu, Fang Xiong, Siyu Ye, Junjun Yang, Haifeng Zhuang, Lili Hong, Shenmeng Gao

**Affiliations:** 1 Medical Research Center, The First Affiliated Hospital of Wenzhou Medical University, 1 Xuefubei Street, Ouhai District, Wenzhou, 325000 Zhejiang Province China; 2 Department of Clinical Laboratory, The First Affiliated Hospital of Wenzhou Medical University, 1 Xuefubei Street, Ouhai District, Wenzhou, Zhejiang Province China; 3 The Children’s Hospital of Zhejiang University School of Medicine, 3333 Binsheng Road, Hangzhou, 310051 Zhejiang Province China; 4 School of Marine Sciences, Ningbo University, 818 Fenghua Road, Jiangbei District, Ningbo, Zhejiang Province China; 5 Department of Laboratory Medicine, The Second Affiliated Hospital, Yuying Children’s Hospital of Wenzhou Medical University, 109 Xuanyuanxi Road, Wenzhou, Zhejiang Province China; 6 Department of Clinical Hematology and Transfusion, The First Affiliated Hospital of Zhejiang Chinese Medical University, 54 Post Road, Hangzhou, Zhejiang Province China; 7 The Key Laboratory of Pediatric Hematology and Oncology Diseases of Wenzhou, the Second Affiliated Hospital, Yuying Children’s Hospital of Wenzhou Medical University, 109 Xuanyuanxi Road, Wenzhou, Zhejiang Province China

**Keywords:** Ferroptosis, Glutathione peroxidase-4, AMPK, Acute myeloid leukemia, Hypomethylating agent

## Abstract

**Background:**

Ferroptosis is a new form of nonapoptotic and iron-dependent type of cell death. Glutathione peroxidase-4 (GPX4) plays an essential role in anti-ferroptosis by reducing lipid peroxidation. Although acute myeloid leukemia (AML) cells, especially relapsed and refractory (R/R)-AML, present high GPX4 levels and enzyme activities, pharmacological inhibition of GPX4 alone has limited application in AML. Thus, whether inhibition of GPX4 combined with other therapeutic reagents has effective application in AML is largely unknown.

**Methods:**

Lipid reactive oxygen species (ROS), malondialdehyde (MDA), and glutathione (GSH) assays were used to assess ferroptosis in AML cells treated with the hypomethylating agent (HMA) decitabine (DAC), ferroptosis-inducer (FIN) RAS-selective lethal 3 (RSL3), or their combination. Combination index (CI) analysis was used to assess the synergistic activity of DAC + RSL3 against AML cells. Finally, we evaluated the synergistic activity of DAC + RSL3 in murine AML and a human R/R-AML-xenografted NSG model in vivo.

**Results:**

We first assessed *GPX4* expression and found that *GPX4* levels were higher in AML cells, especially those with MLL rearrangements, than in NCs. Knockdown of *GPX4* by shRNA and indirect inhibition of GPX4 enzyme activity by RSL3 robustly induced ferroptosis in AML cells. To reduce the dose of RSL3 and avoid side effects, low doses of DAC (0.5 µM) and RSL3 (0.05 µM) synergistically facilitate ferroptosis by inhibiting the AMP-activated protein kinase (AMPK)-SLC7A11-GPX4 axis. Knockdown of *AMPK* by shRNA enhanced ferroptosis, and overexpression of SLC7A11 and GPX4 rescued DAC + RSL3-induced anti-leukemogenesis. Mechanistically, DAC increased the expression of *MAGEA6* by reducing *MAGEA6* promoter hypermethylation. Overexpression of MAGEA6 induced the degradation of AMPK, suggesting that DAC inhibits the AMPK-SLC7A11-GPX4 axis by increasing MAGEA6 expression. In addition, DAC + RSL3 synergistically reduced leukemic burden and extended overall survival compared with either DAC or RSL3 treatment in the MLL-AF9-transformed murine model. Finally, DAC + RSL3 synergistically reduced viability in untreated and R/R-AML cells and extended overall survival in two R/R-AML-xenografted NSG mouse models.

**Conclusions:**

Our study first identify vulnerability to ferroptosis by regulating MAGEA6-AMPK-SLC7A11-GPX4 signaling pathway. Combined treatment with HMAs and FINs provides a potential therapeutic choice for AML patients, especially for R/R-AML.

**Supplementary Information:**

The online version contains supplementary material available at 10.1186/s40164-024-00489-4.

## Background

Acute myeloid leukemia (AML) is characterized by the rapid proliferation of abnormal undifferentiated hematopoietic precursor cells caused by genetic and epigenetic mutations [1]. Although standard chemotherapies induce complete remission in 70–80% of AML patients, most of them will ultimately relapse. Chemotherapy-induced apoptosis is the main form of cell death in AML cells [2, 3]. However, resistance to apoptosis frequently occurs during the clinical treatment of AML patients, resulting in complete treatment failure [4]. Therefore, it is imperative to develop alternative therapeutic strategies to overcome primary and acquired therapy resistance.

Ferroptosis is a new form of nonapoptotic and iron-dependent type of cell death. Glutathione peroxidase-4 (GPX4), a kind of selenoenzyme, is a vital suppressor of ferroptosis that inhibits lipid peroxidation by utilizing glutathione (GSH) [5, 6]. Failure of the lipid peroxide-reducing system caused by genetic ablation or inhibition of GPX4 activity leads to unchecked lipid peroxidation and triggers ferroptosis [5]. Mice with loss of *Gpx4* die during embryonic development [7], suggesting the vital role of GPX4 in anti-lipid peroxidation. Most cancer cells use intracellular cysteine mainly by system xc-mediated uptake of cystine, followed by reduction to cysteine [8]. Glycine, glutamate, and cysteine constitute reduced glutathione (GSH), which is the cofactor of GPX4. Solute carrier family 7 member 11 (SLC7A11) is the transporter subunit in system xc that takes up cystine, and inhibiting SLC7A11 activity by FINs, such as erastin, induces ferroptosis in many cancer cells [9]. Furthermore, ferroptosis is a vital cell death response induced by various chemotherapies, immunotherapies, and radiotherapies [10, 11]. Thus, ferroptosis is a targetable vulnerability of cancer, and targeting ferroptosis by suppressing the SLC7A11-GSH-GPX4 axis may provide new therapeutic methods for treating different cancers. However, whether AML cells depend on the SLC7A11-GSH-GPX4 axis for survival is undetermined.

AMPK is an essential sensor of cellular energy that responds to energy shortages. Once activated by AMP binding or upstream kinase phosphorylation, p-AMPK can phosphorylate downstream targets to reduce ATP-consuming anabolic processes and enhance ATP-generating catabolic processes, restoring energy balance [12]. Several reports have indicated that activation of AMPK facilitates the self-renewal of AML stem cells by regulating mitophagy and glucose transporter 1 [13, 14], suggesting the critical role of AMPK in maintaining leukemogenesis. Although several studies suggest that AMPK activation inhibits ferroptosis [15, 16] or activates ferroptosis [17, 18] in different conditions, whether AMPK regulates ferroptosis in AML cells remains largely unknown.

DNA methylation plays an essential role in leukemogenesis by hypermethylation at the tumor suppressor gene promoter and subsequent silencing of the tumor suppressor gene [19, 20]. Therefore, hypomethylating agents (HMAs), including 5-aza-2-deoxycytidine (decitabine, DAC) and the cytosine analog 5-azacytidine (AZA), have been approved for clinical use in myelodysplastic syndromes (MDS) and AML [21]. HMAs present antileukemia activity in a concentration-dependent manner. HMAs at high doses induce cytotoxicity. HMAs at low doses inhibit DNMT activity and induce DNA hypomethylation, leading to the alteration of the gene expression profile [22, 23]. DNA methylation has emerged as a promising prognostic biomarker in various kinds of cancer because of its high specificity and stability. Furthermore, HMA-based combination therapy with the BCL2 inhibitor venetoclax has become a standard care for older or unfit newly diagnosed AML patients [24]. However, whether HMAs synergize with FINs to trigger ferroptosis in AML cells remains unknown.

Here, we report that low doses of DAC and RSL3 synergistically facilitate ferroptosis in AML cells. DAC increases the expression of MAGEA6 via demethylation of the *MAGEA6* promoter, leading to the degradation of AMPK protein and downregulation of SLC7A11 expression, followed by inhibition of GPX4 enzyme activity. Significantly, DAC and RSL3 synergistically reduce viability in untreated and R/R AML samples, indicating the potential for preclinical use of HMAs + FINs in R/R AML patients.

## Methods

### Cell lines, primary AML blasts, and normal CD34^+^ hematological stem and progenitor cells (HSPCs)

Human leukemic cell lines, including MOLM-13 and MV4-11 (ATCC, Manassas, VA, USA), were incubated in RPMI 1640 with 10% fetal bovine serum (FBS, Invitrogen, Carlsbad, CA, USA) in a humidified 37 °C incubator with 5% CO_2_. Bone marrow (BM) mononuclear cells were isolated by Ficoll density gradient centrifugation (GE Healthcare, Uppsala, Sweden) from AML patients at diagnosis. Primary AML blasts were incubated in StemSpan Serum-Free Expansion Medium (SFEM; Stemcell Technologies, Vancouver, BC, Canada) with recombinant human interleukin-6 (IL-6, PeproTech, Rocky Hill, NJ, USA), stem cell factor (SCF, PeproTech), and interleukin-3 (IL-3, PeproTech) at 10 ng/mL each. Human normal CD34^+^ HSPCs were isolated by the EasySep™ human CD34 positive selection kit (StemCell Technologies) from healthy volunteers and deposited in liquid nitrogen until use. Both AML patients and healthy volunteers provided informed consent according to the Declaration of Helsinki, and all procedures in our studies followed the Declaration of Helsinki and the Ethics Committee of the First Affiliated Hospital of Wenzhou Medical University. The clinical characteristics of the AML patients are summarized in Table S1.

### RNA extraction and quantitative RT-PCR (qRT-PCR)

Total mRNAs from AML cells were extracted by TRIzol reagent (Sigma-Aldrich) according to standard procedures. After extraction, the absorbance at 260/280 nm was measured to assess the concentration and quality of mRNA by a DS-11 spectrophotometer (DeNovix, Wilmington, DE, USA). cDNA for qRT-PCR analysis was synthesized using a Q5 real-time PCR system (Applied Biosystems, Carlsbad, CA, USA). cDNA was reverse transcribed with PrimeScript™ RT Master Mix (Takara Bio, Tokyo, Japan). GAPDH was routinely used as an endogenous control for qRT-PCR. Alternatively, β-actin was used as a loading control as specifically indicated. SYBR Green dye (Takara) was used to determine mRNA expression. Relative expression was calculated using the 2^−ΔΔCT^ method. All of the primer sequences are shown in Table S2.

### Construction of plasmids

Gene-specific short hairpin RNAs (shRNAs) for *GPX4* and *AMPK* were synthesized and inserted into the pLKO.1-puro vector (Clontech, Palo Alto, CA, USA). Control shRNA is a nonfunctional construct. The coding sequences of *MAGEA6* and SLC7A11 were amplified and cloned and inserted into the pLVX-puro vector (Clontech). The PITA-GPX4 vector overexpressing GPX4 was kindly provided by Prof. Tian (Tianjin Medical University). All primer sequences are shown in Table S2, and these constructs were confirmed by DNA sequencing.

### **MLL-AF9-induced murine AML model for** in vivo **treatment**

MSCV-green fluorescent protein (GFP)-internal ribosome entry site (IRES)-MLL-AF9 and the packaging plasmid pCL-ECO were transfected into 293T cells to generate retroviruses. Normal C57/B6 mice (Beijing Vital River Laboratory, Beijing, China) were treated with 5-fluorouracil (5-flu). Mouse Lineage (Lin^−^) cells were isolated after 5-flu treatment and subsequently transduced with MSCV-GFP-IRES-MLL-AF9-retrovirus by spinoculation at 2000×rpm for 2 h [25]. After centrifugation, Lin^−^ cells were incubated in a humidified 37 °C incubator with 5% CO_2_ for another 1 h. Lin^−^ cells were cultured in StemSpan SFEM (Stemcell Technologies) supplemented with murine SCF (100 ng/ml, PeproTech), TPO (100 ng/ml, PeproTech), and FLT3 ligand (100 ng/ml, PeproTech) overnight after centrifugation to remove retroviruses. Furthermore, GFP^+^ Lin^−^ cells were sorted and transplanted into lethally irradiated C57BL/6J mice (Beijing Vital River Laboratory). The recipient mice were humanely sacrificed when they showed signs of paralysis, and BM mononuclear cells were isolated from femur and tibia, and GFP^+^ cells were sorted by flow cytometry. GFP^+^ AML cells (1 × 10^4^/mouse) were xenografted into lethally irradiated C57BL/6J mice, which were randomly divided into four different groups: Ctrl; DAC (2.5 mg/kg), RSL3 (5 mg/kg), and DAC (2.5 mg/kg) + RSL3 (5 mg/kg). RSL3 and DAC were dissolved in solution with 10% DMSO + 45% PEG300 + 45% Saline. The control mice received the placebo (10% DMSO + 45% PEG300 + 45% Saline). Treatment (three times a week for a total of two weeks) was started approximately two weeks after transplantation when the percentage of GFP^+^ cells in peripheral blood (PB) was > 5%. When the Ctrl mice developed full-blown leukemia, the mice were humanely sacrificed by CO_2_ inhalation. BM and PB were isolated to measure the frequency of GFP^+^ cells by flow cytometry. Liver and spleen tissues were subjected to HE staining. All animal procedures and care were performed by national and international policies and institutional guidelines of the ethics committee of the First Affiliated Hospital of Wenzhou Medical University.

### Wright-Giemsa stain

Murine PB smears and BM cytospins were stained by following standard protocols for morphological analysis [26].

### H&E staining

Paraformaldehyde-fixed murine spleen and liver tissue sections were subjected to H&E staining by standard protocols [27].

### DNA methylation detection

To determine the frequency of DNA methylation at the *MAGEA6* promoter, we extracted DNA from AML cells using a DNA Purification Kit (Vazyme Biotech). For sodium bisulfite treatment, the EZ DNA Methylation™-GOLD Kit (ZYMO RESEARCH, Irvine, CA, USA) was used to treat AML cells. The methylation-specific and unmethylation-specific primers were designed by MethPrimer software [28], and methylation-specific PCR (MSP) and unmethylation-specific PCR analysis (UMSP) were performed on sodium bisulfite-treated DNA as a template. For bisulfite-sequencing analysis, sodium bisulfite-treated DNA was used as the template to amplify the CpG island at the *MAGEA6* promoter by PCR. Then, PCR products were subcloned and inserted into the pUC18 vector for direct sequencing. Sodium bisulfite-transformed DNA sequences were subjected to the Quma website to analyze the percentage of methylation in AML cells and NC samples [29].

### Combination index (CI) analysis

CI was calculated by CalcuSyn Software (Biosoft, Cambridge, UK) (CI < 1, synergism; CI > 1, antagonism; CI = 1, additivity). The program was based on the following equation: q = EA + B/(EA + EB-EA × EB), where EA and EB are the inhibition rates of group A and group B, respectively. EA + B is the inhibition rate of group A combined with B [30].

### Other procedures

Chemical Regents, Western blot, CCK-8 assay, Lipid reactive oxygen species (ROS) level assay, Malondialdehyde (MDA) assay, Glutathione (GSH) assay, GPX4 enzyme activity analysis, RNA sequencing (RNA-seq) analysis, Intracellular iron assay, please see supplemental material and methods.

### Statistical analyses

All quantitative results are expressed as the mean ± SD. The significance of the difference between different groups was determined by Student’s t test (two-tailed), one-way ANOVA with Tukey’s multiple comparison test, or two-way ANOVA with Sidak’s multiple comparison test. A *P* value < 0.05 was considered statistically significant. Overall survival (OS) was estimated according to the Kaplan-Meier method. The log-rank test was used to assess the statistical significance of OS. All statistical analyses were performed with Prism version 9.3.0. (*P < 0.05, **P < 0.01, and ***P < 0.001).

## Results

### GPX4 expression is higher in AML cells than in normal controls (NCs)

**Fig. 1:**
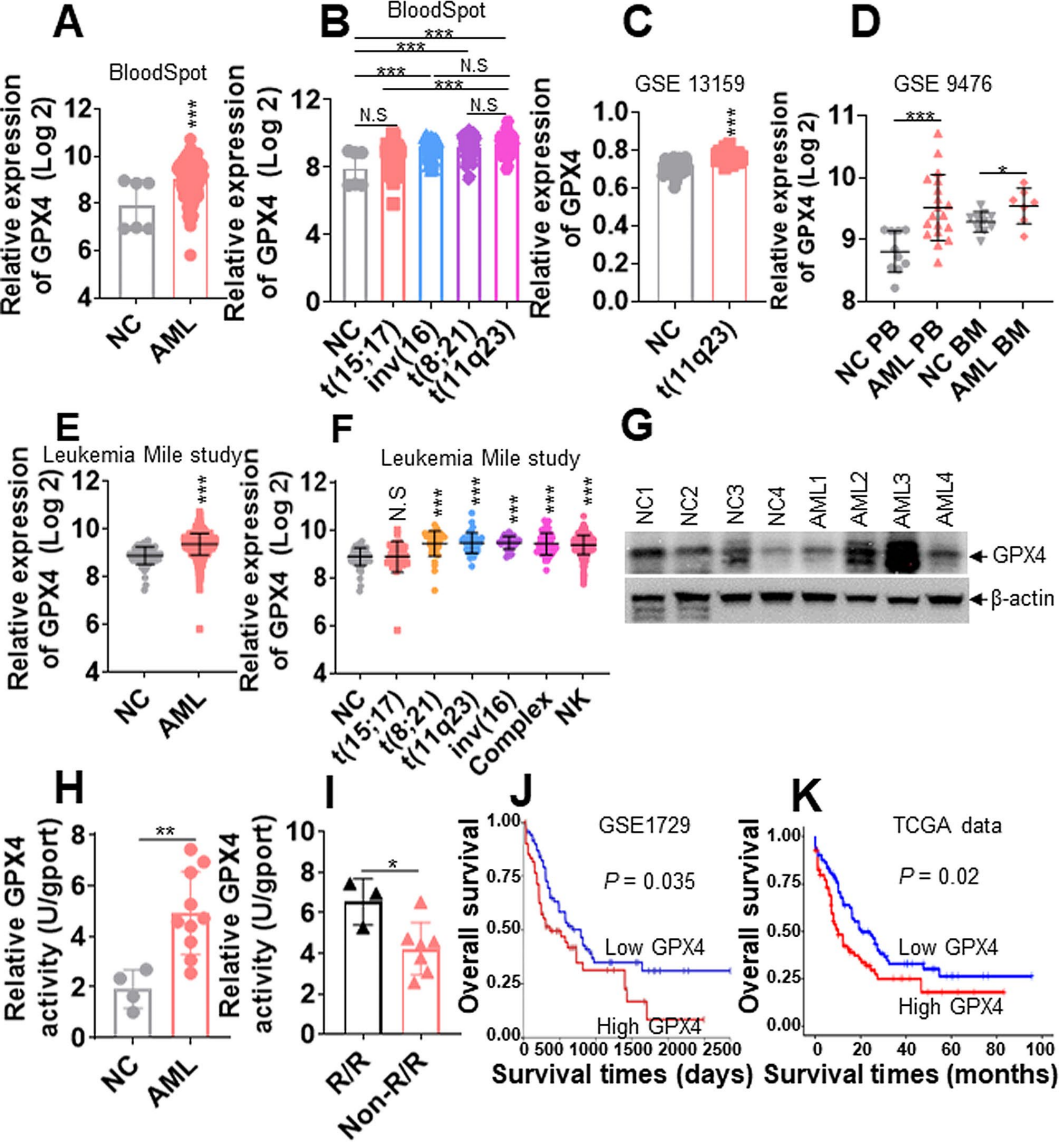
GPX4 expression is higher in AML blasts than in normal controls (NCs). **(A)** The relative expressions of *GPX4* were assessed in AML blasts and normal hematological stem and progenitor cells (HSPCs) as normal controls (NCs) from the BloodSpot database. **(B)** The relative expressions of *GPX4* were determined in AML blasts with different chromosome karyotypes from the BloodSpot database. **(C)** The relative expressions of *GPX4* were assessed in AML cells with t(11q23) arrangement and NCs based on the GSE13159 database. **(D)** The relative expressions of *GPX4* were assessed in PB and BM cells from AML patients and NCs based on the GSE9476 database. **(E)** The relative expressions of *GPX4* were assessed in healthy BM as NC and AML patients from database of Leukemia Mile study. **(F)** The relative expressions of *GPX4* were assessed in different AML karyotype from Leukemia Mile study database. NK: normal karyotype. **(G)** The protein levels of GPX4 were measured in four NCs and four AML samples. **(H)** GPX4 enzyme activities were measured in four NCs and ten AML samples. **(I)** GPX4 enzyme activities were analyzed in three R/R-AML and seven non-R/R-AML cells. (**J** and **K**) The overall survival of AML patients with higher expression of *GPX4* (above median) and lower expression of *GPX4* (below median) was assessed in the GSE1729 **(J)** and TCGA databases **(K)**. *P < 0.05; **P < 0.01; ***P < 0.001 versus NCs. N.S: not significant

Several reports have demonstrated that tumor cells enhance ferroptosis resistance by increasing GPX4 expression or enhancing enzyme activity [31, 32]. To explore the potential function of GPX4 in AML blasts, we first assessed the level of *GPX4* in AML cells from the BloodSpot database [33] and found that *GPX4* expression was higher in AML cells than in normal HSPCs as NCs (Fig. 1A). In addition, *GPX4* was highly expressed in AML cells with t(8;21) carrying the AML1-ETO fusion gene, t(11q23) bearing MLL rearrangements, and inv(16) bearing CBFβ-MYH11 but not those with t(15;17) carrying PML-RARα (Fig. 1B). *GPX4* expression was highest in AML patients carrying t(11q23) rearrangements compared with those bearing t(8;21), inv(16), or t(15;17) rearrangements (Fig. 1B). It was statistically significant (*P* < 0.001) between t(11q23) and t(15;17) rearrangement. However, there was no statistically significant between t(11q23) and t(8;21) rearrangement (*P* > 0.05) or between t(11q23) and inv(16) rearrangement (*P* > 0.05) (Fig. 1B). Furthermore, *GPX4* expression was higher in AML cells with t(11q23) rearrangement than in NCs based on the GSE13159 database (Fig. 1C). In addition, GPX4 expression was higher in PB and BM from AML cells than in those from NCs according to the GSE9476 database (Fig. 1D). Furthermore, we analyzed *GPX4* levels from Leukemia Mile study [34], and found that *GPX4* levels were higher in AML samples than in NCs (Fig. 1E). GPX4 levels were also higher in AML cells with t(8;21), t(11q23), inv(16), complex karyotype, and normal karyotype (NK) compared with NCs (Fig. 1F). However, GPX levels were similar in AML cells with t(15;17) than in NCs (Fig. 1F). Our studies have demonstrated that *GPX4* transcripts were overexpressed in AML cells compared with NCs. We further measured the protein levels of GPX4 and found that GPX4 protein levels were higher in four AML blasts (Table S1) than in four NCs on average (Fig. 1G).

Although *GPX4* transcript and protein levels were higher in AML cells compared with NC, whether GPX4 enzyme activity is also higher in AML cells than in NCs is undetermined. We measured GPX4 enzyme activity in lysates from 4 NCs and 10 AML cells (Table S1) and found that GPX4 enzyme activities were approximately 2.6-fold higher in AML blasts than in NCs (Fig. 1H). Because R/R cells are more resistance to apoptosis or ferroptosis than non-R/R AML cells, we then determined whether R/R AML blasts have higher GPX4 enzyme activity. We analyzed GPX4 enzyme activity in three R/R AML blasts and seven non-R/R cells (Table S1). GPX4 enzyme activities were higher in R/R AML blasts than in non-R/R cells (Fig. 1I).

To finally explore the effects of GPX4 on the overall survival of AML patients, we analyzed the overall survival in GSE1729 and TCGA datasets. AML patients with higher expression of *GPX4* (above median) were associated with lower overall survival in comparison to those with lower expression of *GPX4* (below median) based on GSE1729 (Fig. 1J) and TCGA database (Fig. 1K). Thus, our results demonstrate that AML cells, especially R/R-AML cells, have higher GPX4 level and enzyme activity compared with NC samples. Higher expression of GPX4 predicts poorer outcomes in AML patients than in those with lower levels of GPX4.

### **Knockdown of*GPX4*and inhibition of GPX4 activity induce ferroptosis** in vitro

**Fig. 2:**
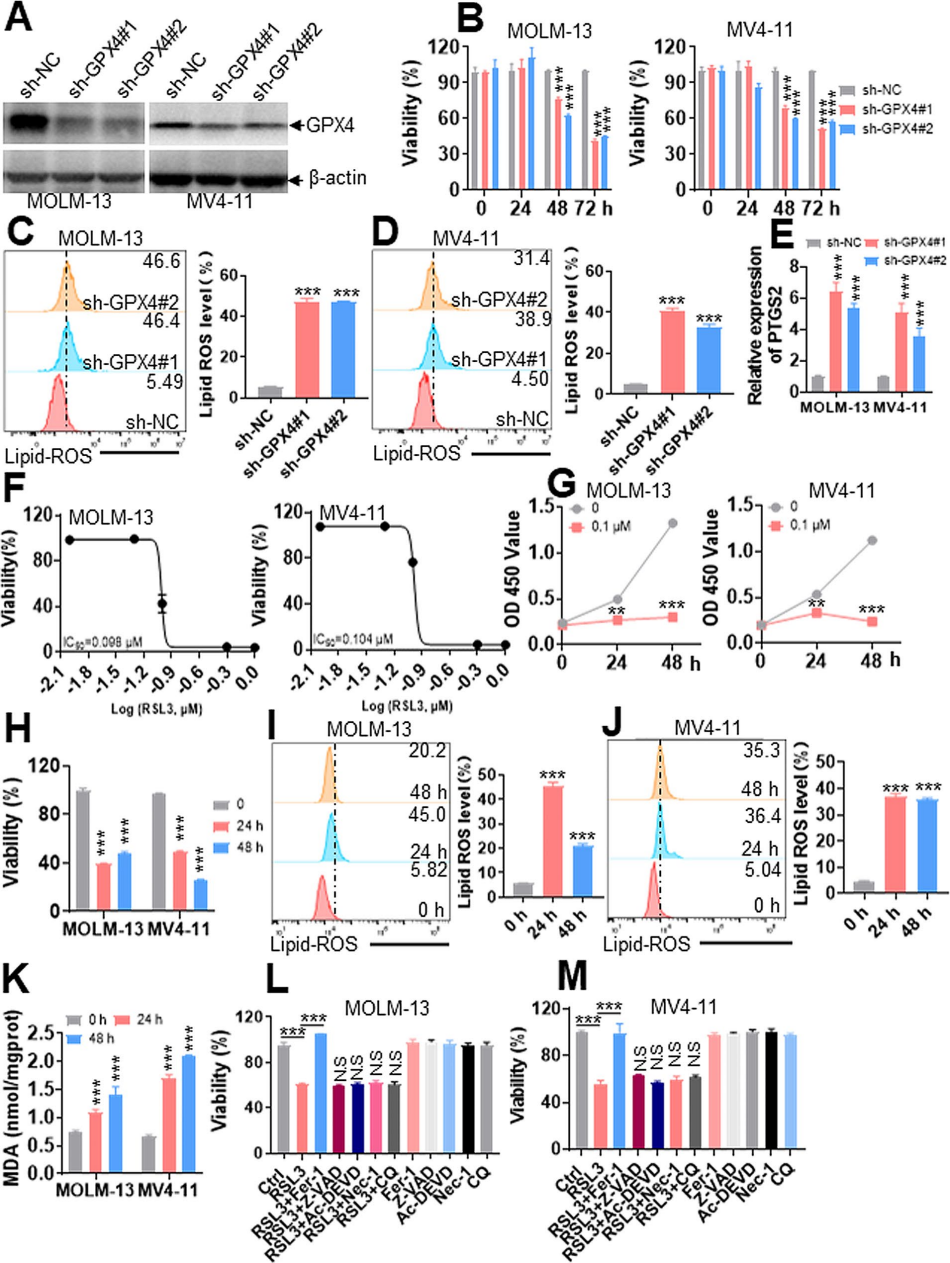
Knockdown of *GPX4* and indirect inhibition of GPX4 activity by RSL3 trigger ferroptosis in AML cells. **(A)** MOLM-13 and MV4-11 cells were transduced with two specific shRNAs for *GPX4* (sh-GPX4) or negative control (sh-NC) to inhibit *GPX4* expression, and the protein levels of GPX4 were measured. **(B)** Viability was measured in MOLM-13 and MV4-11 cells after transduction with sh-GPX4 or sh-NC for 24, 48, and 72 h. (**C** and **D**) Lipid ROS levels were measured by flow cytometry in MOLM-13 and MV4-11 cells with knockdown of *GPX4* or NC. Representative plots (left) and statistical analysis of lipid ROS levels (right) are shown. **(E)** Relative *PTGS2* levels were measured in MOLM-13 and MV4-11 cells after transduction with sh-GPX4 or sh-NC. **(F)** Cell viability was measured by CCK-8 assay in MOLM-13 and MV4-11 cells treated with the indicated concentrations of RSL3 for 24 h, and the IC50 was calculated. (**G** and **H**) Cell proliferation and viability were measured in MOLM-13 and MV4-11 cells treated with or without RSL3 (0.1 µM) for 24 and 48 h. (**I** and **J**) Lipid ROS levels were measured in MOLM-13 and MV4-11 cells treated with RSL3 (0.1 µM) for 24 and 48 h. Shown are the representative plots (left) and statistical analysis of lipid ROS levels (right). **(K)** MDA amounts were measured in MOLM-13 and MV4-11 cells treated with or without RSL3 (0.1 µM) for 24 and 48 h. (**L** and **M**) Viability was measured in MOLM-13 and MV4-11 cells, which were preincubated with Fer-1 (2 µM), Z-VAD (20 µM), Ac-DEVD (20 µM), CQ (10 µM), or Nec-1 (50 µM) for 1 h and then treated with RSL3 (0.1 µM) for 24 h. **P < 0.01; ***P < 0.001 versus negative control or untreated cells. N.S (not significant) compared with RSL3 treatment

As reported, GPX4 presents anti-ferroptosis ability by reducing lipid peroxidation [5]. We determined whether *GPX4* depletion can induce ferroptosis in AML cells. Because *GPX4* expression is highest in AML cells with (t11q23) carrying MLL rearrangements (Fig. 1B), we selected MOLM-13 and MV4-11 cells carrying MLL rearrangements for the following experiments. Transduction of specific shRNA for *GPX4* efficiently reduced the protein expression of GPX4 in AML cells (Fig. 2A). As expected, *GPX4* deficiency substantially reduced cell viability (Fig. 2B), triggered lipid ROS level (Fig. 2C-D), and increased the expression of *PTGS2* (Fig. 2E), a marker of ferroptosis [5], in MOLM-13 and MV4-11 cells.

To further explore the role of GPX4 in anti-ferroptosis activity, we selected RSL3, a widely used GPX4 inhibitor, to assess the function of GPX4 inhibition in AML cells [5]. We first measured the IC50 of RSL3 and found that it was approximately 0.1 µM in MOLM-13 and MV4-11 cells at 24 h (Fig. 2F). Consequently, RSL3 (0.1 µM) treatment substantially reduced cell proliferation and inhibited viability at 24 and 48 h (Fig. 2G-H). We then measured intracellular lipid ROS levels and MDA amounts, two well-accepted markers during ferroptosis. RSL3 treatment triggered lipid ROS levels (Fig. 2I-J) and increased MDA amounts at 24 and 48 h (Fig. 2K). To determine whether RSL3 induced ferroptosis but not apoptosis, autophagy, or necroptosis, we used the ferroptosis inhibitor Fer-1, the apoptosis inhibitors Z-VAD and Ac-DEVD, autophagy inhibitor chloroquine (CQ), and the necroptosis inhibitor necrostatin-1 (Nec-1) to explore the activity of RSL3 against AML cells. Fer-1, but not Z-VAD, Ac-DEVD, CQ, or Nec-1, almost rescued the decreased viability caused by RSL3 treatment (Fig. 2L-M), suggesting that RSL3 induces ferroptosis but not apoptosis, autophagy, or necroptosis.

### HMAs and FINs synergistically trigger ferroptosis in AML cells

**Fig. 3:**
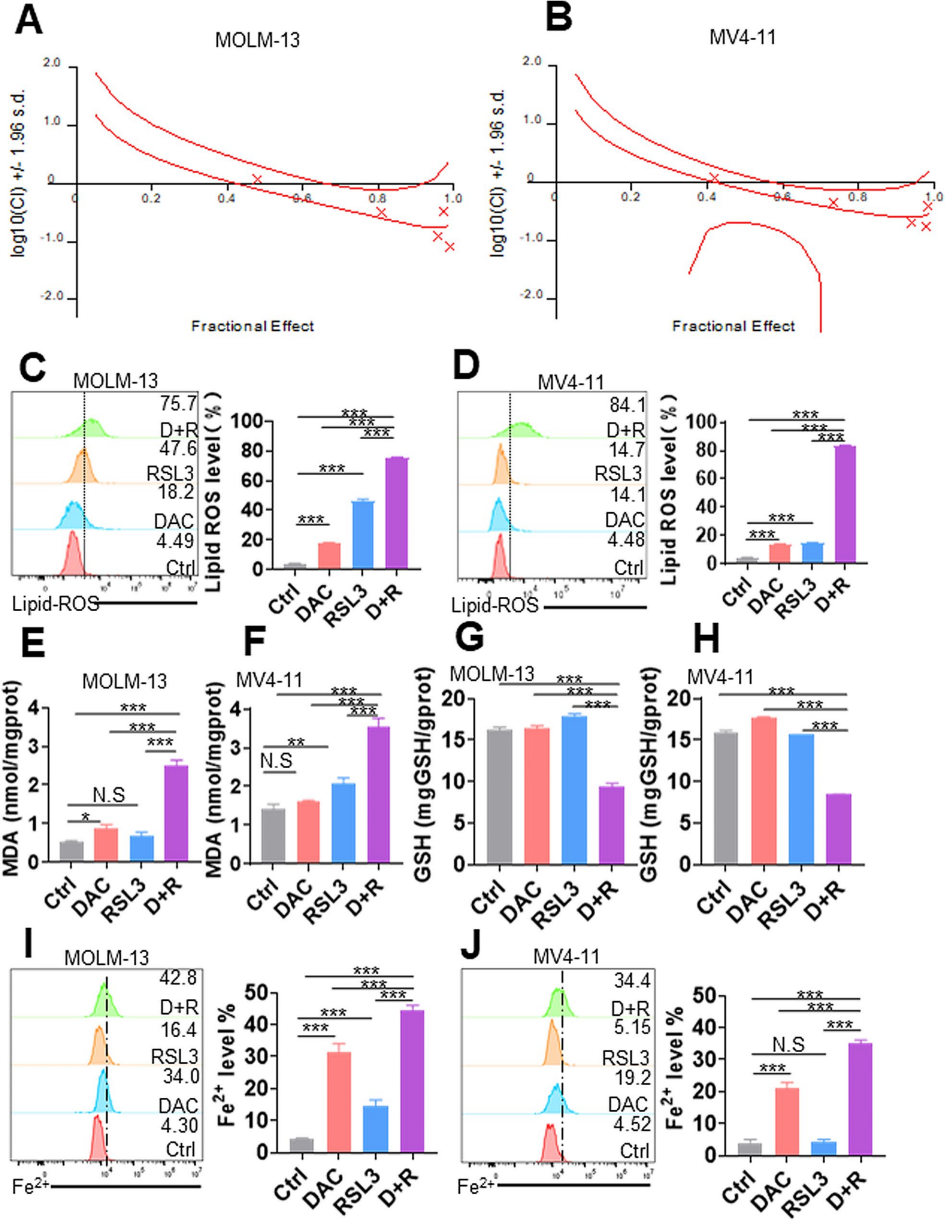
DAC and RSL3 synergistically induce ferroptosis in AML cells in vitro. (**A** and **B**) AML cells were treated with different concentrations of DAC (0.25 µM, 0.5 µM, 0.75 µM, 1.0 µM, 2.5 µM) for 48 h, RSL3 (0.025 µM, 0.05 µM, 0.075 µM, 0.1 µM, 0.25 µM) for 24 h, or their combination. CalcuSyn was used to assess the possible synergistic effects. CI < 1.0 is considered a synergistic effect. (**C** and **D**) Lipid ROS levels were measured in MOLM-13 and MV4-11 cells treated with DAC (0.5 µM) for 48 h, RSL3 (0.05 µM) for 24 h, or their combination. Representative plots (left) and statistical analysis of lipid ROS levels (right) are shown. (**E**–**H**) MDA (**E** and **F**) and GSH amounts (**G** and **H**) were measured in MOLM-13 and MV4-11 cells treated with DAC, RSL3, or DAC + RSL3 cotreatment. (**I** and **J**) The intracellular iron assay was performed by flow cytometry in MOLM-13 and MV4-11 cells treated with Ctrl, DAC, RSL3, or DAC + RSL3 cotreatment. Representative plots (left) and statistical analysis of Fe^2+^ levels (right) are shown. *P < 0.05; **P < 0.01; ***P < 0.001. N.S: not significant

Recently, inducing ferroptosis with FINs has been a promising therapeutic strategy to eliminate cancers [35]. However, single FIN, such as RSL3, has limited clinical application because of low water solubility and potential side effects [36]. Therefore, FINs combined with other drugs might improve antileukemia outcomes. Since HMA-based combination therapy with BCL2 inhibitor notably improves the outcome in AML patients [24], we determined whether FINs combined with HMAs have synergistic activity against AML. AML cells were treated with DAC, RSL3 and their combinations at various concentrations, and cell viability was measured. CalcuSyn was used to assess the possible synergistic effects. Four different concentration combinations of RSL3 and DAC had a CI < 1.0 in MOLM-13 and MV4-11 cells (Fig. 3A-B and Fig. S1A-B), demonstrating the robust synergistic activity of DAC + RSL3 against AML cells. Thus, low doses of DAC (0.5 µM) and RSL3 (0.05 µM) were used for the following experiments. DAC + RSL3 significantly reduced cell viability compared with either of the two agents alone (Fig. S1C-D). Then, lipid ROS levels, MDA amounts, GSH amounts, and Fe^2+^ staining were measured in AML cells treated with DAC + RSL3 or either of the two agents alone. The combination treatment synergistically increased lipid ROS levels (Fig. 3C-D), upregulated MDA amounts (Fig. 3E-F), decreased GSH amounts (Fig. 3G-H), and increased Fe^2+^ staining levels (Fig. 3I-J) in comparison to single DAC or RSL3 treatment. Finally, Fer-1 but not Z-VAD, CQ, or Nec-1 almost rescued the decreased viability by DAC + RSL3 treatment (Fig. S2A-B), suggesting that DAC + RSL3 induces ferroptosis, but not apoptosis, autophagy, or necroptosis.

We wanted to further confirm that DAC had synergistic activity with more FINs in addition to RSL3. AML cells were treated with DAC and the SLC7A11 inhibitor erastin [9]. As expected, DAC + erastin significantly reduced cell viability (Fig. S3A-B) and increased lipid ROS levels compared with either of the two agents alone (Fig. S3E-F). Furthermore, cotreatment of DAC with another GPX4 inhibitor FIN56 [37] also notably decreased cell viability (Fig. S3C-D) and triggered lipid ROS levels compared with either of the two agents alone (Fig. S3G-H).

### DAC regulates the AMPK-SLC7A11-GPX4 signaling pathway to enhance ferroptosis

**Fig. 4:**
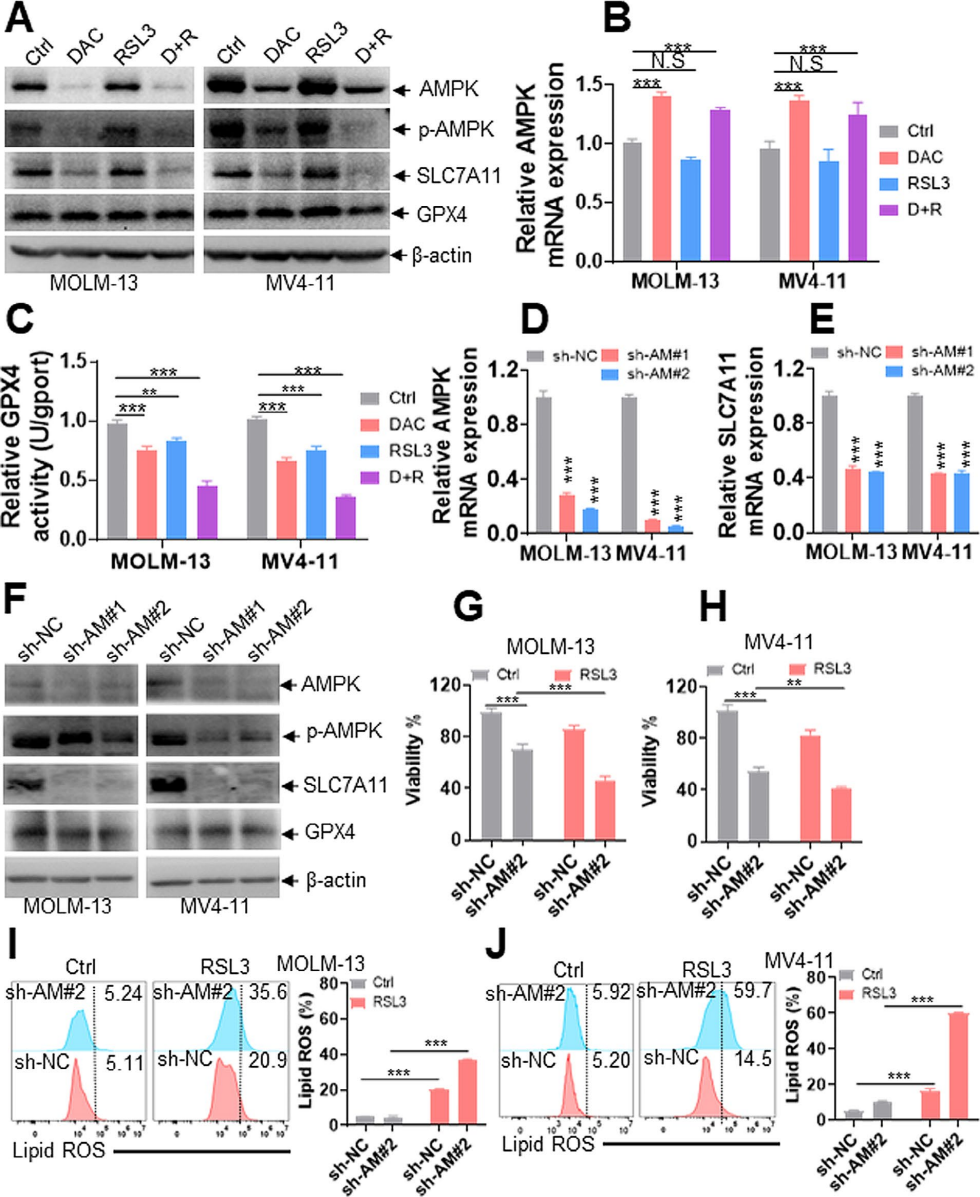
DAC enhances RSL3-induced ferroptosis by regulating the AMPK-SLC7A11-GPX4 signaling pathway. **(A)** AMPK, p-AMPK, SLC7A11, and GPX4 protein levels were measured in MOLM-13 and MV4-11 cells treated with DAC (0.5 µM) for 48 h, RSL3 (0.05 µM) for 24 h, or their combination DAC (0.5 µM) + RSL3 (0.05 µM) (D + R). (**B** and **C**) AMPK transcript level **(B)** and GPX4 enzyme activity **(C)** were measured in MOLM-13 and MV4-11 cells treated with Ctrl, DAC, RSL3, or D + R cotreatment. (**D** and **E**) *AMPK* and *SLC7A11* transcripts were measured in MOLM-13 and MV4-11 cells transduced with shRNAs for AMPK (sh-AM#1 and #2) or negative control (sh-NC). (**F**) AMPK, p-AMPK, SLC7A11, and GPX4 protein levels were measured in MOLM-13 and MV4-11 cells transduced with sh-AM or sh-NC. (**G** and **H**) Viability was measured in MOLM-13 and MV4-11 cells transduced with sh-AM#2 or sh-NC, which were further treated with RSL3 (0.05 µM) or Ctrl for 24 h. (**I** and **J**) Lipid ROS levels were measured in MOLM-13 and MV4-11 cells transduced with sh-AM#2 or sh-NC, which were further treated with RSL3 or Ctrl for 24 h. **P < 0.01; ***P < 0.001. N.S: not significant

Our study has demonstrated that DAC facilitates SLC7A11 inhibitor- and GPX4 inhibitor-induced ferroptosis. AMPK, an important cellular energy sensor, mediates ferroptosis, probably through the SLC7A11-GPX4 signaling pathway [15, 38]. We then explored whether DAC enhances ferroptosis though the AMPK-mediated SLC7A11-GPX4 axis. AMPK, p-AMPK, SLC7A11, and GPX4 protein levels were first measured in leukemic cells treated with Ctrl, DAC, RSL3, or DAC + RSL3. DAC and DAC + RSL3 substantially reduced the protein expressions of AMPK, p-AMPK, and SLC7A11 (Fig. 4A) but did not affect the GPX4 protein level (Fig. 4A). However, RSL3 treatment did not reduce the protein expressions of AMPK, p-AMPK, SLC7A11, and GPX4 (Fig. 4A). Furthermore, DAC treatment moderately increased the *AMPK* transcript level (Fig. 4B), suggesting that DAC-induced AMPK protein degradation might be independent of transcript synthesis. Because DAC + RSL3 did not substantially affect GPX4 protein level, we measured GPX4 enzyme activity. DAC + RSL3 notably inhibited GPX4 enzyme activity compared to either of the two agents alone (Fig. 4C).

In addition, AZA, another widely used HMA, significantly reduced the protein expressions of AMPK, p-AMPK, and SLC7A11 (Fig. S4A). We further explored whether AZA had synergistic activity with RSL3 against AML cells. AZA + RSL3 substantially decreased viability (Fig. S4B-C) and increased the lipid ROS level compared with AZA or RSL3 treatment alone (Fig. S4D-E). These results confirm that HMAs inhibit the AMPK-SLC7A11-GPX4 signaling pathway and have synergistic activity with FINs to facilitate ferroptosis.

To further determine the role of AMPK in DAC + RSL3-induced ferroptosis, AML cells were treated with the specific AMPK inhibitor compound C. Compound C notably decreased SLC7A11 protein expression (Fig. S5A). In addition, compound C + RSL3 significantly reduced cell viability compared with either of them (Fig. S5B). In contrast, the specific AMPK activator A-769,662 increased SLC7A11 protein expression (Fig. S5A). To exclude the possible off-target effects of AMPK activator or inhibitor, *AMPK* expression was inhibited by two specific shRNAs. Two shRNAs caused an apparent decrease in *AMPK* and *SLC7A11* transcripts (Fig. 4D-E) and a noticeable reduction in AMPK, p-AMPK, and SLC7A11 protein expressions (Fig. 4F).

To determine whether *GAPDH* could be an appropriate loading control for qRT-PCR because AMPK axis is responsible for metabolic regulation. We measured *β-actin* transcript level and analyzed the expression of *GAPDH* using *β-actin* as a loading control in AML cells with knockdown of *AMPK* or not. *AMPK* knockdown did not affect *GAPDH* expression when *β-actin* was used a loading control (Fig. S6A). Also, *AMPK* mRNA expression was decreased by two specific shRNAs than negative control (sh-NC) using *β-actin* as a loading control (Fig. S6B).

Furthermore, MOLM-13 and MV4-11 cells with knockdown of *AMPK* were treated with RSL3. Knockdown of *AMPK* by shRNA or RSL3 treatment resulted in a moderate decrease in viability (Fig. 4G-H), while *AMPK* knockdown + RSL3 substantially reduced cell viability compared with *AMPK* knockdown or RSL3 treatment alone (Fig. 4G-H). More importantly, *AMPK* knockdown + RSL3 substantially increased lipid ROS levels compared with *AMPK* knockdown or RSL3 treatment alone (Fig. 4I-J).

### **Overexpression of SLC7A11 or GPX4 rescues DAC + RSL3-induced antileukemia effect**

**Fig. 5:**
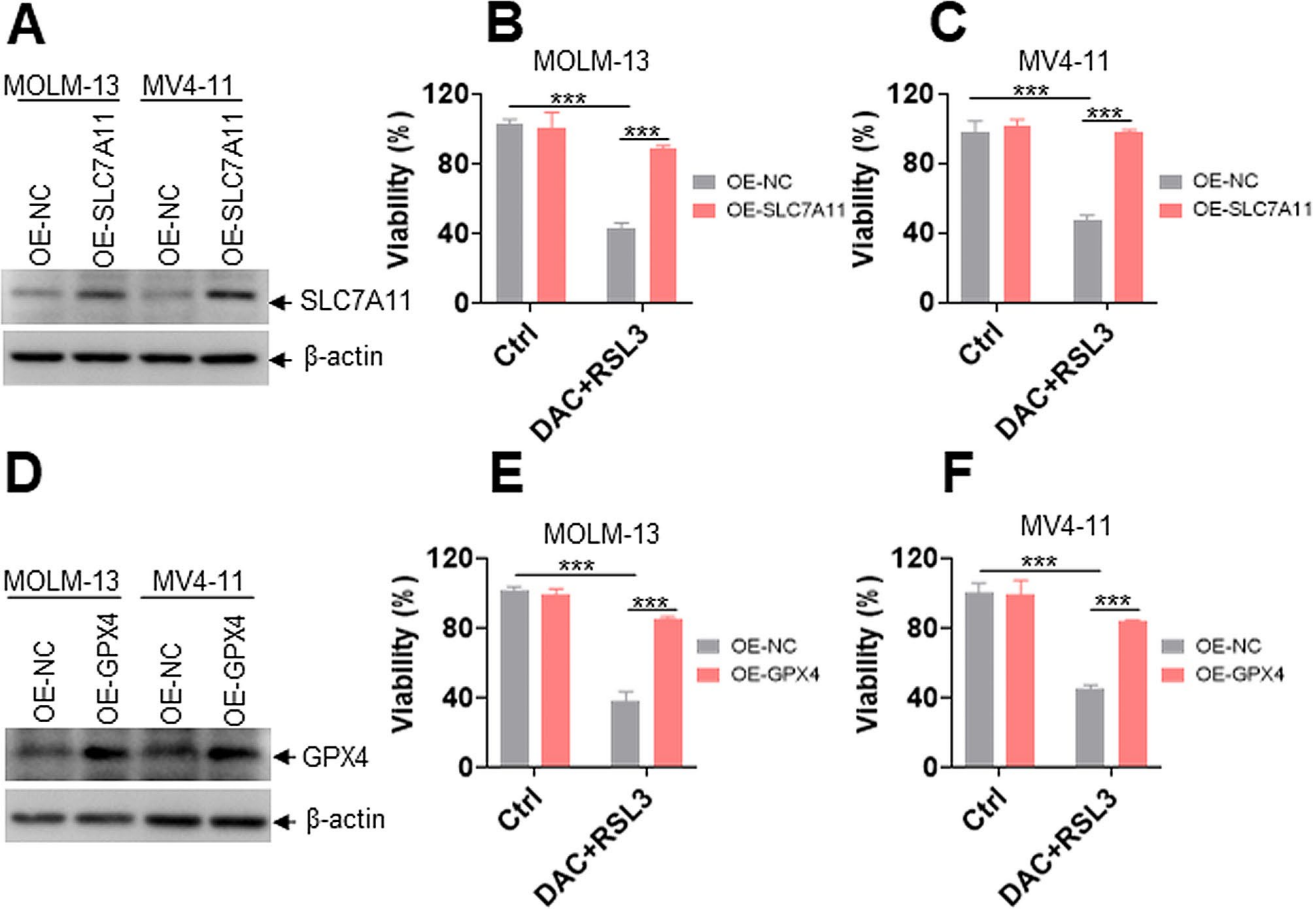
Overexpression of SLC7A11 or GPX4 rescues DAC + RSL3-induced decrease in viability. **(A)** Western blot was performed in MOLM-13 and MV4-11 cells, which were overexpressed with SLC7A11 or NC. (**B** and **C**) Viability was measured in Ctrl- or DAC + RSL3-treated MOLM-13 and MV4-11 cells, which were further overexpressed with SLC7A11 or NC. **(D)** Western blot was performed in MOLM-13 and MV4-11 cells, which were overexpressed with GPX4 or NC. (**E** and **F**). Viability was measured in Ctrl or DAC + RSL3-treated MOLM-13 and MV4-11 cells overexpressing GPX4 or NC. ***P < 0.001

Because DAC + RSL3 treatment reduced the expression of SLC7A11 and weakened GPX4 enzyme activity, we further determined whether overexpression of SLC7A11 or GPX4 can rescue the antileukemia effect of DAC + RSL3. Western blot analysis demonstrated that SLC7A11 was successfully overexpressed in AML cells (Fig. 5A), and overexpression of SLC7A11 markedly rescued the decreased viability induced by DAC + RSL3 (Fig. 5B-C). Furthermore, overexpression of GPX4 (Fig. 5D) almost completely prevented the DAC + RSL3-induced decrease in viability (Fig. 5E-F).

### DAC induces AMPK degradation by regulating MAGEA6 promoter hypermethylation

**Fig. 6:**
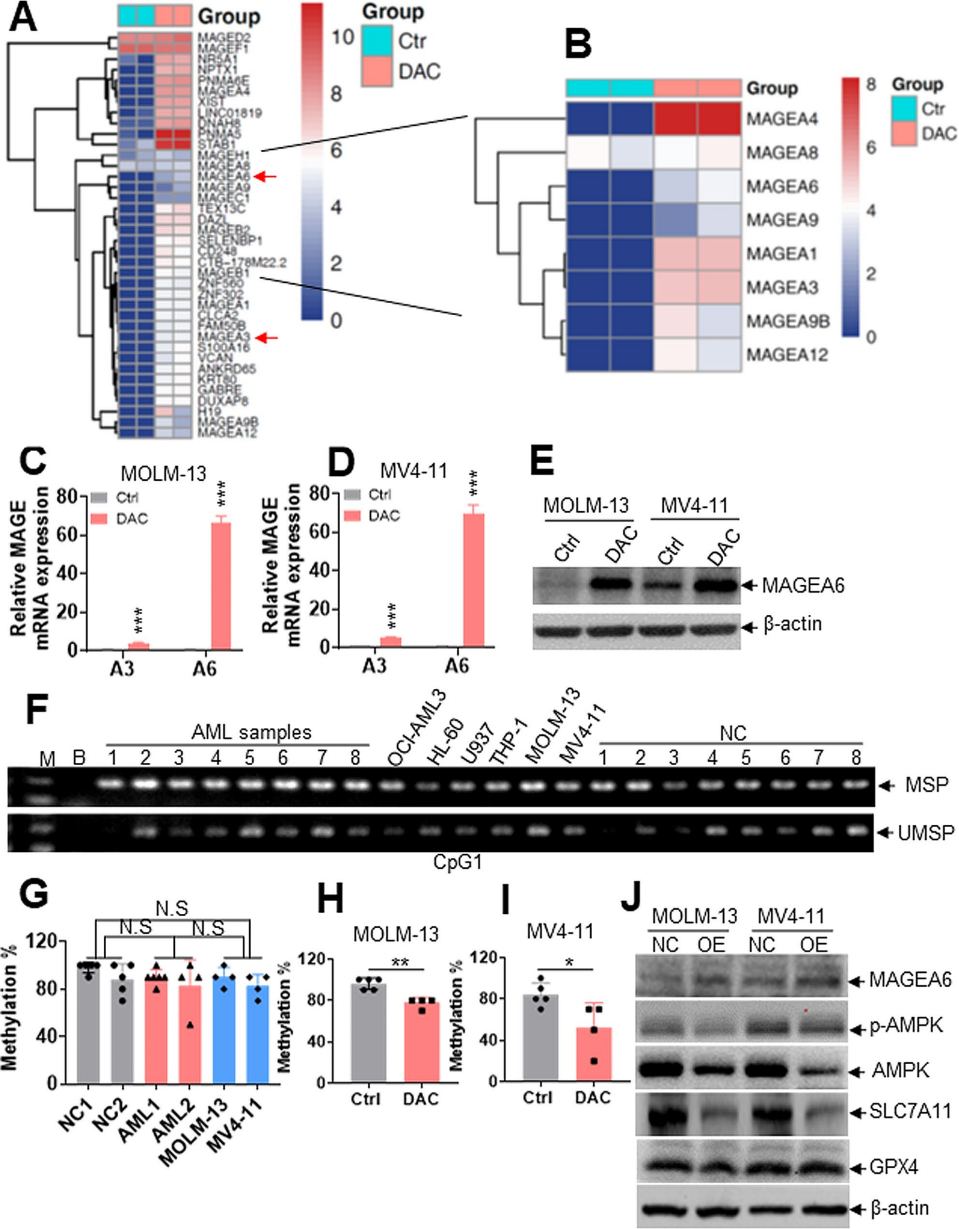
DAC induces AMPK degradation by increasing MAGEA6 expression and reducing MAGEA6 promoter hypermethylation. (**A** and **B**) RNA-seq analysis of DAC- and Ctrl-treated MOLM-13 cells. (**C** and **D**) *MAGEA3* and *A6* transcript levels were measured in MOLM-13 and MV4-11 cells treated with or without DAC (0.5 µM) for 48 h. **(E)** MAGEA6 protein level was measured in MOLM-13 and MV4-11 cells treated with DAC (0.5 µM) for 48 h. **(F)** Methylation-specific PCR (MSP) and unmethylation-specific PCR (UMSP) were performed to measure the methylation level of CpG island 1 in eight AML samples, six AML cell lines, and eight NCs. M: DNA Marker; B: Blank. **(G)** Bisulfite-genomic sequencing was performed to measure the methylation status of CpG island 1 in two NCs, two AML samples, and two AML cell lines. (**H** and **I**) MOLM-13 and MV4-11 cells were incubated with or without DAC (0.5 µM) for 48 h. DNA was extracted for bisulfite-genomic sequencing in Ctrl- or DAC-treated AML cells, and a summary of the frequencies of methylated CpG dinucleotides is shown. **(J)** The protein expression levels of MAGEA6, p-AMPK, AMPK, SLC7A11, and GPX4 were measured in MOLM-13 and MV4-11 cells transduced with pLVX-MAGEA6 overexpressing MAGEA6 (OE) or blank vector pLVX-NC (NC). *P < 0.05; **P < 0.01; ***P < 0.001 versus Ctrl cells. N.S: not significant

Because DAC treatment decreased the AMPK protein level but slightly increased the *AMPK* transcript level, we speculate that the ubiquitin-proteasome system might mediate AMPK degradation by DAC. To finally elucidate the underlying mechanism of AMPK degradation by DAC, RNA-seq was performed in Ctrl and DAC-treated cells. Interestingly, the transcript levels of *MAGE* family members, such as *MAGEA3* and *A6*, were notably increased in DAC-treated cells compared with the Ctrl group (Fig. 6A-B). MAGE family members are silenced in normal tissues except in the testis but are aberrantly re-expressed in cancer tissues [39]. As reported, MAGEA3 and A6 mediate the degradation of AMPK via the ubiquitin-proteasome system [40, 41]. Therefore, we explored whether DAC treatment induces the degradation of AMPK by increasing MAGEA3/6 expressions. DAC treatment notably increased the expressions of *MAGEA3/6* in AML cells (Fig. 6C-D), although the increased extent of *MAGEA6* is higher than that of *MAGEA3*. Thus, we focused on the function of MAGEA6. As expected, DAC treatment markedly increased MAGEA6 protein expression (Fig. 6E). To further investigate whether *MAGEA6* promoter methylation mediates its expression, we first used MethPrimer software to determine possible CpG islands in the *MAGEA6* promoter. Two CpG islands were found in the putative *MAGEA6* promoter (Fig. S7A). Using MSP and UMSP, we found that six AML cell lines and eight primary AML blasts (Table S1) presented heavy methylation in CpG island 1 (Fig. 6F). However, NC samples also had a high methylation status (Fig. 6F). Bisulfite-sequencing analysis was performed further to determine the exact methylation level in the *MAGEA6* promoter. The methylation level in the *MAGEA6* promoter was similarly high between two AML cell lines, two primary AML blasts, and two NC samples (Fig. 6G and Fig. S7B). Most importantly, DAC treatment decreased methylation levels compared with Ctrl treatment by Bisulfite-sequencing analysis (Fig. 6H-I and Fig. S7C-D). Furthermore, using MSP and UMSP analysis, we found that DAC treatment weakened methylation but enhanced unmethylation at *MAGEA6* promoter in MOLM-13 and MV4-11 cells than Ctrl group (Fig. S7E). Thus, DAC treatment increases MAGEA6 expression by reducing the methylation level at the *MAGEA6* promoter. Finally, we explored whether MAGEA6 facilitates AMPK protein degradation [40]. Overexpression of MAGEA6 markedly induced AMPK protein degradation, followed by the inhibition of p-AMPK and the reduction of SLC7A11 protein level (Fig. 6J). However, overexpression of MAGEA6 did not affect GPX4 protein level in AML cells (Fig. 6J).

### **DAC + RSL3 has synergistic activity in an MLL-AF9-transformed murine model** in vivo

**Fig. 7:**
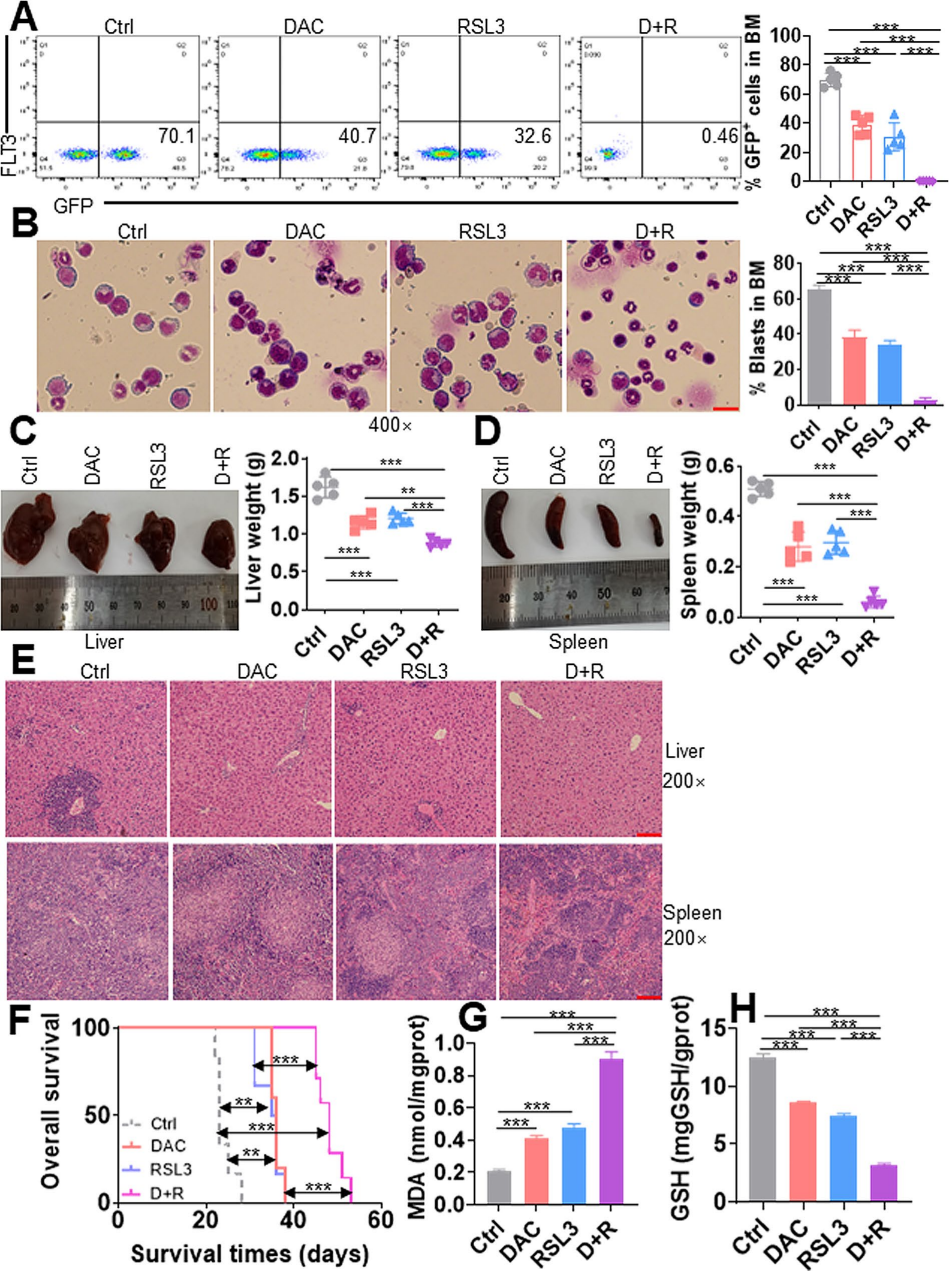
DAC and RSL3 synergistically exert antileukemic activity in the MLL-AF9-transformed murine AML model. **(A)** The frequencies of GFP^+^ cells were measured in BM mononuclear cells from Ctrl, DAC, RSL3, or DAC + RSL3 (D + R)-treated AML mice (*n* = 4 for each group). Representative plots (left) and statistical analysis of GFP^+^ cells (right) are shown. **(B)** The Wright-Giemsa stain showed BM blasts from Ctrl, DAC, RSL3, or D + R-treated AML mice. Representative pictures (left) and statistical analysis of the percentage of BM blasts (right) are shown. Bar scales represent 20 μm. (**C** and **D**) Liver and spleen tissues were isolated from Ctrl, DAC, RSL3, or D + R-treated AML mice (*n* = 4 for each group), and weights were calculated. Representative pictures (left) and statistical analysis of the liver and spleen weights (right) are shown. **(E)** Representative images of HE staining of liver and spleen tissues from Ctrl, DAC, RSL3, or D + R-treated leukemic mice. Bar scales represent 100 μm for spleen and liver tissues. **(F)** Overall survival was calculated in Ctrl (*n* = 6), DAC (*n* = 5), RSL3 (*n* = 6), or D + R (*n* = 7)-treated leukemic mice. (**G** and **H**) MDA **(G)** and GSH amounts **(H)** were measured in BM GFP^+^ cells isolated from AML mice treated with Ctrl, DAC, RSL3, or D + R (*n* = 3 for each group). **P < 0.01; ***P < 0.001

To further investigate the antileukemia activity of DAC + RSL3, we used an MLL-AF9-transformed murine model for this test in vivo [42]. Murine GFP^+^ AML cells were transplanted into recipient mice, which were treated with DAC + RSL3, DAC, RSL3, or vehicle as the Ctrl after transplantation for two weeks (Fig. S8A). We first measured the percentage of GFP^+^ cells representing AML blasts in PB and BM. Although single DAC or RSL3 treatment decreased GFP^+^ cells in PB (Fig. S8B) and BM cells (Fig. 7A) to a certain extent. DAC + RSL3 almost completely eradicated leukemic cells and significantly decreased the percentage of GFP^+^ cells compared with DAC or RSL3 treatment alone in PB (Fig. S8B) and BM cells (Fig. 7A). Wright-Giemsa staining demonstrated that leukemic blasts of PB (Fig. S8C) and BM (Fig. 7B) cells were markedly reduced in DAC + RSL3-cotreated mice compared with single DAC- or RSL3-treated mice. To further determine whether DAC + RSL3 reduced the infiltration of leukemic cells in liver and spleen tissues, we measured the weight of liver and spleen tissues. Although DAC and RSL3 mono treatment decreased liver weight by approximately 30% (Fig. 7C) and spleen weight by approximately 50% (Fig. 7D) compared to the Ctrl group, respectively, DAC + RSL3 almost decreased liver weight by approximately 50% (Fig. 7C) and spleen weight by 80% (Fig. 7D) compared to single DAC or RSL3 treatment. Furthermore, HE staining demonstrated that DAC + RSL3 almost completely eradicated leukemic cells in liver and spleen tissues compared with single DAC or RSL3 treatment (Fig. 7E). Finally, DAC + RSL3 markedly extended overall survival compared with either of the two agents alone (Fig. 7F).

To determine whether the effects of DAC + RSL3 against the murine AML model were caused by ferroptosis, MDA and GSH amounts were measured in BM GFP^+^ AML cells. As expected, DAC + RSL3 markedly increased MDA amounts but decreased GSH amounts compared with single DAC or RSL3 treatment in murine AML cells (Fig. 7G-H).

### DAC + RSL3 has synergistic activity in primary AML blasts but not in normal HSPCs

**Fig. 8:**
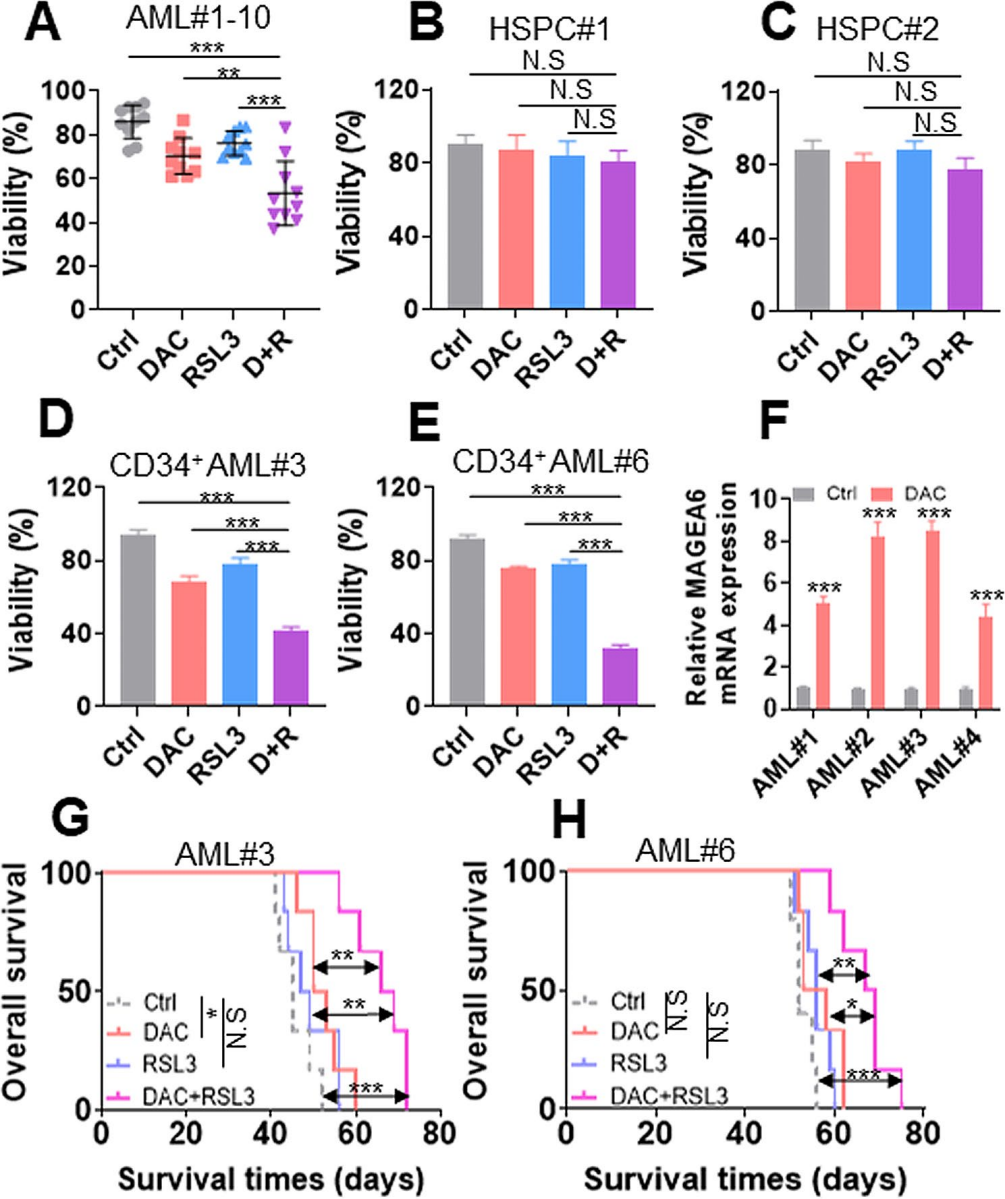
DAC and RSL3 synergistically have antileukemic effects in primary AML samples but not in normal HSPCs. **(A)** Cell viability was measured in 10 AML samples treated with Ctrl, DAC (1.0 µM) for 48 h, RSL3 (0.1 µM) for 24 h, or their combination in vitro. Each scatter plot represents mean value of one AML patient. (**B** and **C**) Cell viability was measured in two normal CD34^+^ HSPCs treated with Ctrl, DAC (1.0 µM) for 48 h, RSL3 (0.1 µM) for 24 h, or their combination. (**D** and **E**) Cell viability was measured in two CD34^+^ cells, which were isolated from two R/R AML patients, treated with Ctrl, DAC (1.0 µM) for 48 h, RSL3 (0.1 µM) for 24 h, or their combination. **(F)** *MAGEA6* transcript levels were measured in four primary AML blasts treated with Ctrl or DAC (1.0 µM) for 48 h in vitro. (**G** and **H**) Overall survival was calculated in Ctrl (*n* = 6 for G and *n* = 5 for H), DAC (*n* = 6 for **G** and **H**), RSL3 (*n* = 6 for G and H), or DAC + RSL3 (*n* = 6 for G and H)-treated NSG mice transplanted with R/R AML cells. *P < 0.05; **P < 0.01; ***P < 0.001. N.S: not significant

To further determine the role of DAC + RSL3 in primary AML blasts, cell viability was measured in 10 AML samples, including seven untreated AML cells and three R/R AML samples (Table S1), which were treated with DAC + RSL3 or either of them. DAC + RSL3 substantially reduced cell viability compared with either of the two agents alone in 8 of 10 AML samples (Fig. 8A and Fig. S9A-J). Most importantly, DAC + RSL3 presented synergistic antileukemia ability in three R/R AML samples (Fig. S9C, F and J). However, DAC + RSL3 and either of the two agents alone did not reduce the viability of the two normal CD34^+^ HSPCs (Fig. 8B-C). We further isolated CD34^+^ cells from primary AML# 3 and #6, followed by treatment with DAC + RSL3 or either of them. As expected, DAC + RSL3 markedly reduced cell viability compared with either of the two agents alone in two CD34^+^ AML cells (Fig. 8D-E). To explore whether DAC treatment increased the expression of MAGEA6 in primary AML cells, four AML samples (AML#1–4) were treated with DAC, and *MAGEA6* transcripts were measured. DAC treatment increased the expression of *MAGEA6* in all four AML samples (Fig. 8F). Finally, NSG mice were xenografted with two R/R-AML blasts (AML#3 and #6) and treated with DAC + RSL3 or either of them. DAC + RSL3 substantially prolonged overall survival compared with either single treatment in two R/R-AML-transplanted mice (Fig. 8G-H).

## Discussion

**Fig. 9:**
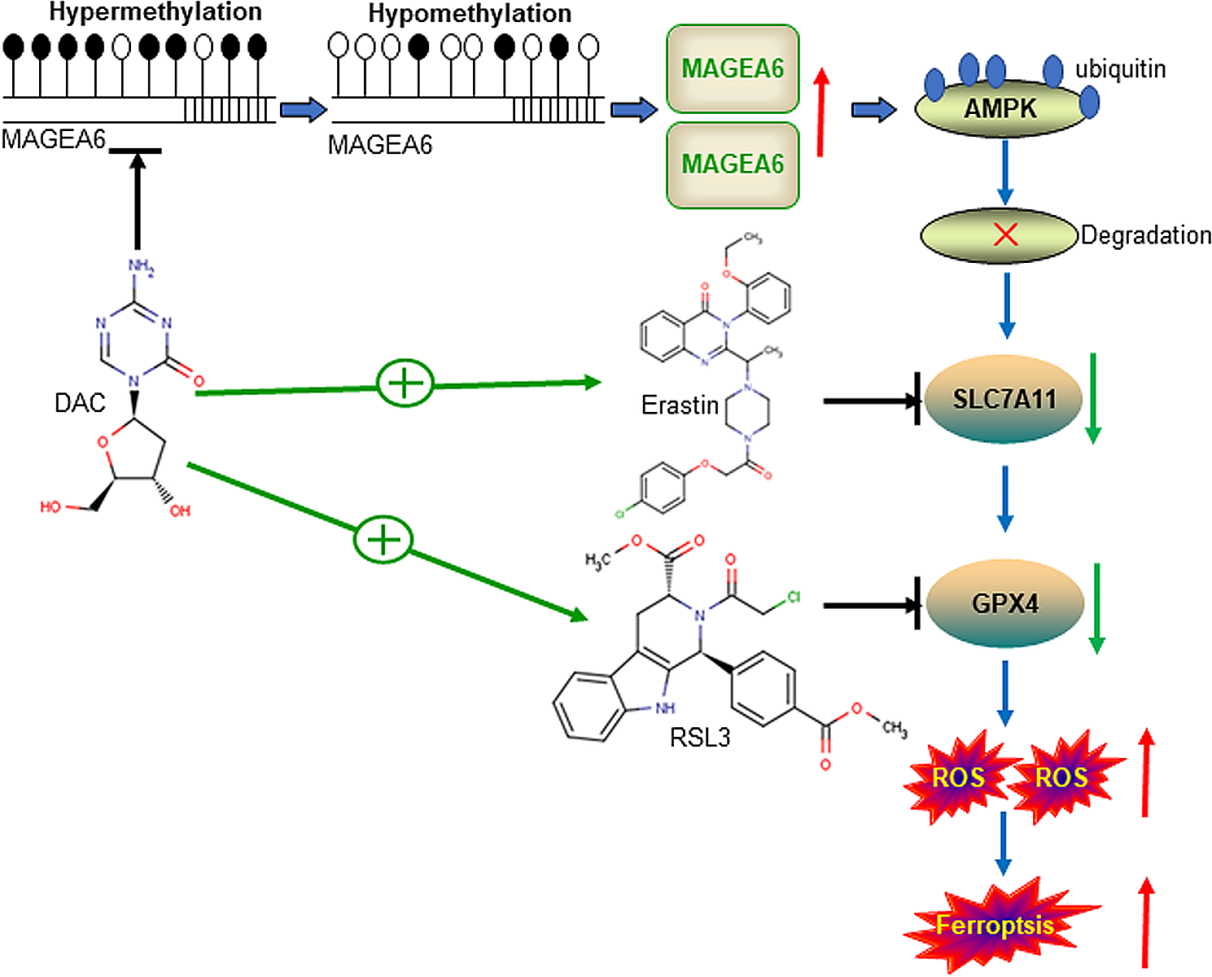
Mechanistic scheme underlying the synergistic activity of DAC with erastin/RSL3. DAC increases MAGEA6 expression by reducing the frequency of methylation at the *MAGEA6* promoter. Overexpression of MAGEA6 induces the degradation of AMPK protein and the inhibition of AMPK activation, leading to the decreased expression of SLC7A11 and attenuated GPX4 enzyme activity. Thus, DAC synergizes with erastin/RSL3 to augment ferroptosis in AML cells

Here, we first report that HMAs with FINs synergistically enhance ferroptosis in AML cells compared with HMA or FIN treatment alone. DAC increases MAGEA6 expression, which is silenced in AML cells by DNA hypermethylation. Increased expression of MAGEA6 induces the degradation of AMPK, leading to decreased expression of SLC7A11 and attenuated GPX4 enzyme activity. Therefore, DAC with erastin/RSL3 synergistically augments ferroptosis in AML cells (Fig. 9). Our study provides a new therapeutic choice for AML patients, especially R/R-AML.

The SLC7A11-GSH-GPX4 signaling pathway has been reported as the primary anti-ferroptosis system in cancer cells [43]. Pardieu B et al. reported that higher expression of SLC7A11 is associated with poor outcome in AML patients, and genetic and chemical inhibition of SLC7A11 triggers ferroptosis and reduces viability [44], suggesting that cystine uptake inhibition might be a front-line therapy in AML. In addition, anthracycline daunorubicin with the SLC7A11 inhibitor sulfasalazine synergistically augments ferroptosis [44]. Additionally, the PARP inhibitor olaparib synergizes with FINs by inhibiting SLC7A11 in BRCA-proficient ovarian cancer [45]. Consistent with these reports, we first found that DAC cooperates with the SLC7A11 inhibitor erastin to facilitate ferroptosis. Mechanistically, DAC treatment decreases AMPK protein level, leading to a reduced level of SLC7A11. Furthermore, knockdown of *AMPK* by shRNA reduces SLC7A11 expression. Thus, DAC augments ferroptosis by regulating the AMPK-SLC7A11 axis. However, the precise molecular mechanism by which AMPK regulates SLC7A11 expression remains unknown.

The antioxidant enzyme GPX4 is the downstream gene of SLC7A11, and GPX4 converts phospholipid hydroperoxides to lipid alcohols, inhibiting ferroptosis [46]. GPX4 is highly expressed in AML blasts to confront high intracellular ROS levels. Genetic ablation by shRNA and indirect inhibition of GPX4 activity by RSL3 trigger ferroptosis in AML cells, suggesting that GPX4 is required for the survival of AML and that GPX4 is a potential target for AML. Our results demonstrate that DAC does not reduce the protein expression of GPX4 but inhibits GPX4 enzyme activity, probably because DAC reduces SLC7A11 levels to limit cystine uptake, leading to reduced GSH amount. As a cofactor of GPX4, the decreased GSH level inhibits GPX4 enzyme activity. Thus, DAC synergizes with RSL3 to augment ferroptosis by regulating the SLC7A11-GSH-GPX4 axis.

Although Yang et al. reported that RSL3 directly binds to GPX4 protein and inactivates the peroxidase activity of GPX4 via unbiased chemoproteomic approach [5], the subsequent report indicated that RSL3 is not a direct inhibitor of GPX4 but of TXNRD1 [47]. These contradictory results suggest that it is difficult and complicated to explore the binding of chemical compounds to protein. However, most reports still indicated that RSL3 is generally considered as GPX4 inhibitor at least through indirectly inhibiting GPX4 enzyme activity [48–50]. Our results demonstrated that low concentration of RSL3 did not affect GPX4 protein level (Fig. 4A), but inhibited GPX4 enzyme activity (Fig. 4C). Therefore, we speculate that RSL3 induces ferroptosis probably through indirectly inhibiting GPX4 enzyme activity, but not decrease GPX4 protein level.

Several reports have demonstrated that AMPK activation is needed for the maintenance of leukemia and the stemness of leukemia-initiating cells [13, 14, 51]. However, the exact molecular mechanism by which AMPK maintains leukemogenesis has not been determined. Because ferroptosis is a vital tumor suppression mechanism [31], we propose that AMPK activation-mediated ferroptosis inhibition might be required to survive AML cells. Our results demonstrate that AMPK inhibition by compound C decreased and its activation by A-769,662 increased SLC7A11 protein expression, supporting a negative regulatory role of AMPK in regulating ferroptosis in AML cells. Additionally, reports indicate that energy stress-mediated AMPK activation and LKB1-AMPK axis activation inhibit ferroptosis [15, 52]. Our reports also demonstrate that knockdown of *AMPK* by shRNA enhances ferroptosis. Thus, triggering ferroptosis by AMPK inhibition may eliminate leukemia cells. However, Song et al. found a promoting role of AMPK in regulating ferroptosis [17], which is inconsistent with our results described here. We speculate that the function of AMPK in regulating ferroptosis might be context dependent, and further investigation is needed.

Although MAGEA6 is only expressed in the testis, we found that MAGEA6 is expressed at a moderate level in AML cells, which is consistent with reports that MAGEA6 is re-expressed in cancer cells [53, 54]. One primary regulatory mechanism to control MAGEA6 expression is promoter CpG methylation in somatic cells [55]. However, the exact methylated sites were still undetermined before our study. We found two CpG islands at the *MAGEA6* promoter and measured the accurate methylated sites in CpG island 1. AML cells present hypermethylation at the *MAGEA6* promoter by MSP and bisulfite-sequencing analysis. DAC treatment reduces the methylation level by approximately 15% and increases the transcript and protein levels of MAGEA6 in AML cells, suggesting that promoter methylation modulates the silencing of MAGEA6 expression. These results are consistent with reports that DAC treatment decreases 5-methylcytosine content by approximately 10.16–59.46% and induces MAGEA family gene expression [56, 57]. Interestingly, HSPC samples also present hypermethylation at the *MAGEA6* promoter. We speculated that MAGEA6 re-expression in AML cells is attributable to epigenetic deregulation, such as active histone modifications [58].

Our results confirm that AML cells and NC samples both present hypermethylation at the *MAGEA6* promoter by MSP and bisulfite-sequencing analysis. As reported, AMPK is constitutively active in AML leukemia initiating cells (LICs) or leukemia stem cells (LSCs), and genetic depletion of AMPK in primary AML cells profoundly suppresses leukemogenesis [13, 14]. Thus, AMPK is more active in LICs/LSCs than NC samples. Therefore, we speculate that DAC treatment preferentially inhibits the activated AMPK signaling pathway by increasing MAGEA6 expression and subsequent degradation of p-AMPK and AMPK in AML cells. In contrast, DAC treatment might have less effect on AMPK signaling pathway in NC samples, which have less AMPK activation.

The survival rate for R/R-AML patients is extremely poor, and new treatments are urgently needed [59]. The therapeutic resistance of cancer cells circumvents death by inhibiting ferroptosis. For example, the lymphatic environment protects metastasizing melanoma cells to enhance their survival rate by weakening ferroptosis [60]. However, mesenchymal cancer cells, often resistant to various treatments, are highly sensitive to ferroptosis by targeting GPX4 [61]. Thus, triggering ferroptosis has anticancer activity in cancer cells with therapy resistance. However, whether R/R-AML cells are vulnerable to ferroptosis has not been determined. Our results first demonstrate that DAC + RSL3 synergistically reduces cell viability in three R/R-AML samples and extends overall survival in two R/R-AML-xenografted NSG mouse model. As reported, more leukemia stem cells (LSCs) are enriched in R/R-AML cells, and LSCs express higher p-AMPK levels than non-LSCs [13]. Therefore, DAC + RSL3 probably exerts antileukemia effects by reducing p-AMPK levels in primary R/R AML cells. However, our sample size is small, and more R/R AML samples are needed for further study.

Recently, Zhao et al. reported that human hematological stem cells (HSCs), not progenitors, are vulnerable to ferroptosis [62]. However, only RSL3 concentrations (> 0.25 µM) induce ferroptosis in human HSCs [62]. We found that low-dose RSL3 (0.1 µM) cooperated with DAC (1.0 µM) to reduce viability in primary AML cells. Correspondingly, DAC and RSL3 at such low concentrations did not reduce viability in human HSPCs. Therefore, low-dose DAC (1.0 µM) and RSL3 (0.1 µM) are relatively safe for further application in AML treatment.

The level and activity of SLC7A11 are precisely modulated at multiple levels, including transcription and posttranslational modifications. For example, NRF2 regulates transcription by directly binding to the SLC7A11 promoter [63]. The primary limitation of our study is that we cannot elucidate the molecular mechanism by which AMPK regulates SLC7A11 expression. Knockdown of *AMPK* by shRNA decreased both mRNA and protein expression of SLC7A11, indicating that AMPK might reduce the synthesis of *SLC7A11* mRNA or decrease the stability of *SLC7A11* mRNA, finally leading to the reduced expression of SLC7A11 protein.

## Conclusions

In this report, we first identify the vulnerability to ferroptosis by regulating MAGEA6-AMPK-SLC7A11-GPX4 signaling pathway in AML cells. HMAs synergize with FINs to facilitate ferroptosis by increasing MAGEA6 expression and subsequent inhibiting the AMPK-SLC7A11-GPX4 signaling pathway. Although R/R AML cells present high GPX4 enzyme activity, they are vulnerability to ferroptosis induced by HMAs + FINs cotreatment. Therefore, HMAs + FINs provide a possible therapeutic choice for AML, especially R/R-AML patients, through triggering ferroptosis.

### Electronic supplementary material

Below is the link to the electronic supplementary material.

Supplementary Material 1

Supplementary Material 2

Supplementary Material 3

Supplementary Material 4

Supplementary Material 5

Supplementary Material 6

Supplementary Material 7

Supplementary Material 8

Supplementary Material 9

Supplementary Material 10

Supplementary Material 11

Supplementary Material 12

Supplementary Material 13

## Data Availability

The datasets used and/or analyzed during the current study are available from the corresponding author upon reasonable request.

## Abbreviations

GPX4: Glutathione peroxidase 4
AML: Acute leukemia myeloid
FIN: Ferroptosis inducer
RSL3: RAS-selective lethal 3
HMA: Hypomethylating agent
DAC: Decitabine
AMPK: AMP-activated protein kinase
AZA: 5-azacytidine
MAGEA6: Melanoma antigen A6
GSH: Glutathione
SLC7A11: Solute carrier family 7 member 11
MDS: Myelodysplastic syndromes
qRT-PCR: quantitative RT-PCR
CI: Combination index
ROS: Reactive oxygen species
MDA: Malondialdehyde
OS: Overall survival
R/R-AML: Relapsed and refractory AML
HSPCs: Hematopoietic stem progenitor cells
