# Supplementary figures and images for: Low-dose hypomethylating agents cooperate with ferroptosis inducers to enhance ferroptosis by regulating the DNA methylation-mediated MAGEA6-AMPK-SLC7A11-GPX4 signaling pathway in acute myeloid leukemia
Source: Exp Hematol Oncol. 2024 Feb 20;13:19. doi: 10.1186/s40164-024-00489-4 (PMC10877917; doi:10.1186/s40164-024-00489-4)

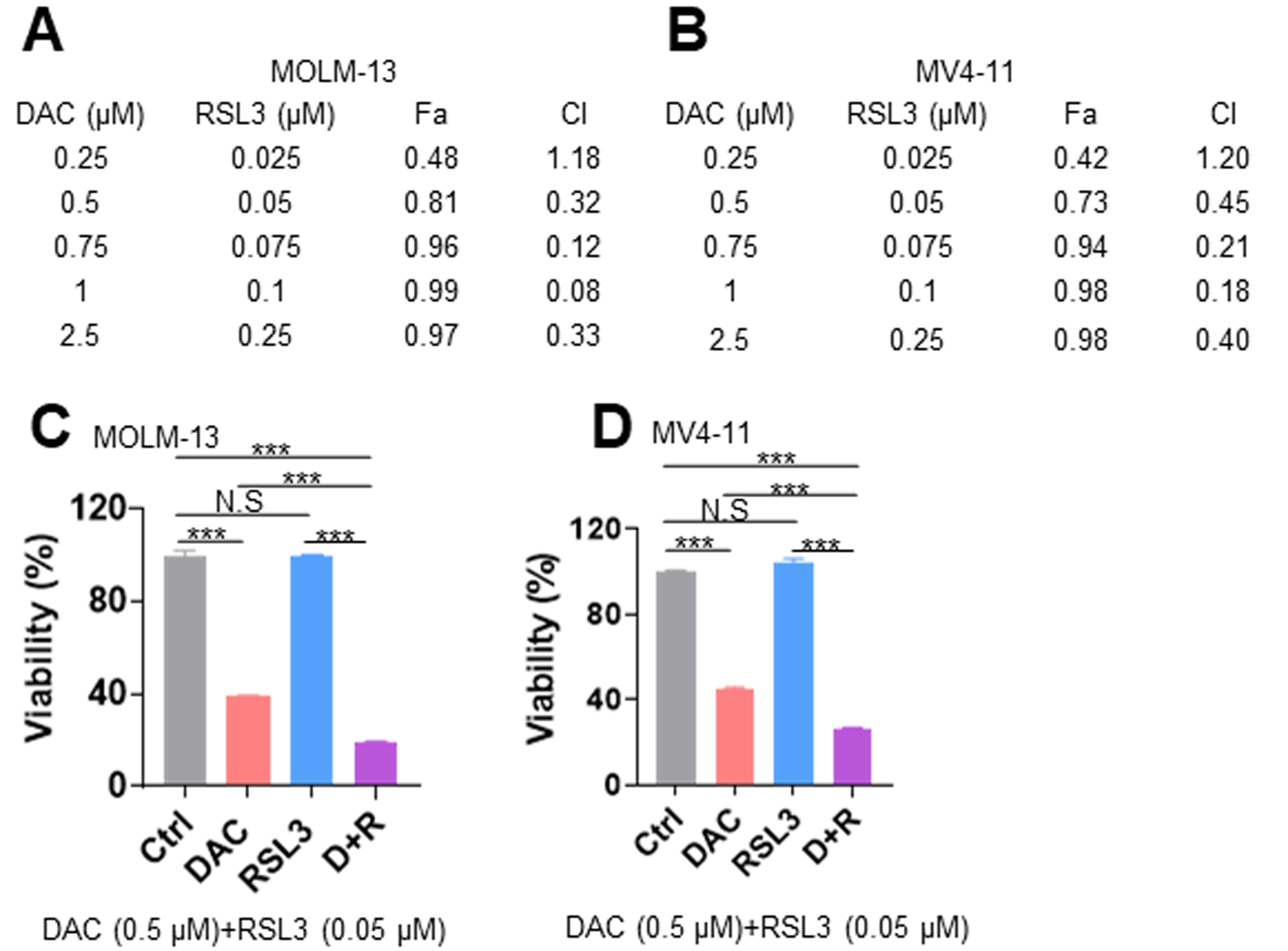

Supplement: Supplementary file 1 — Supplementary Material 1 [file 40164_2024_489_MOESM1_ESM.tif]

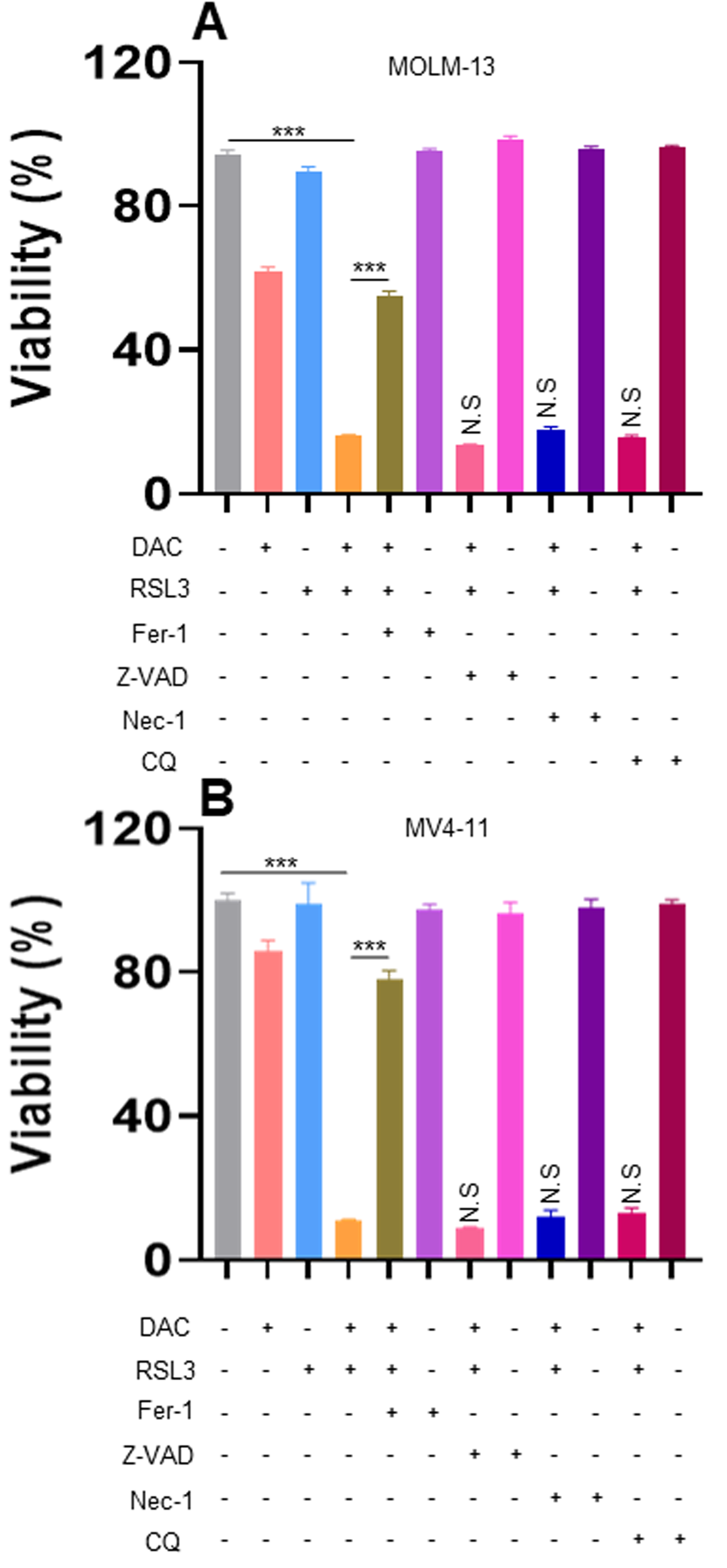

Supplement: Supplementary file 2 — Supplementary Material 2 [file 40164_2024_489_MOESM2_ESM.tif]

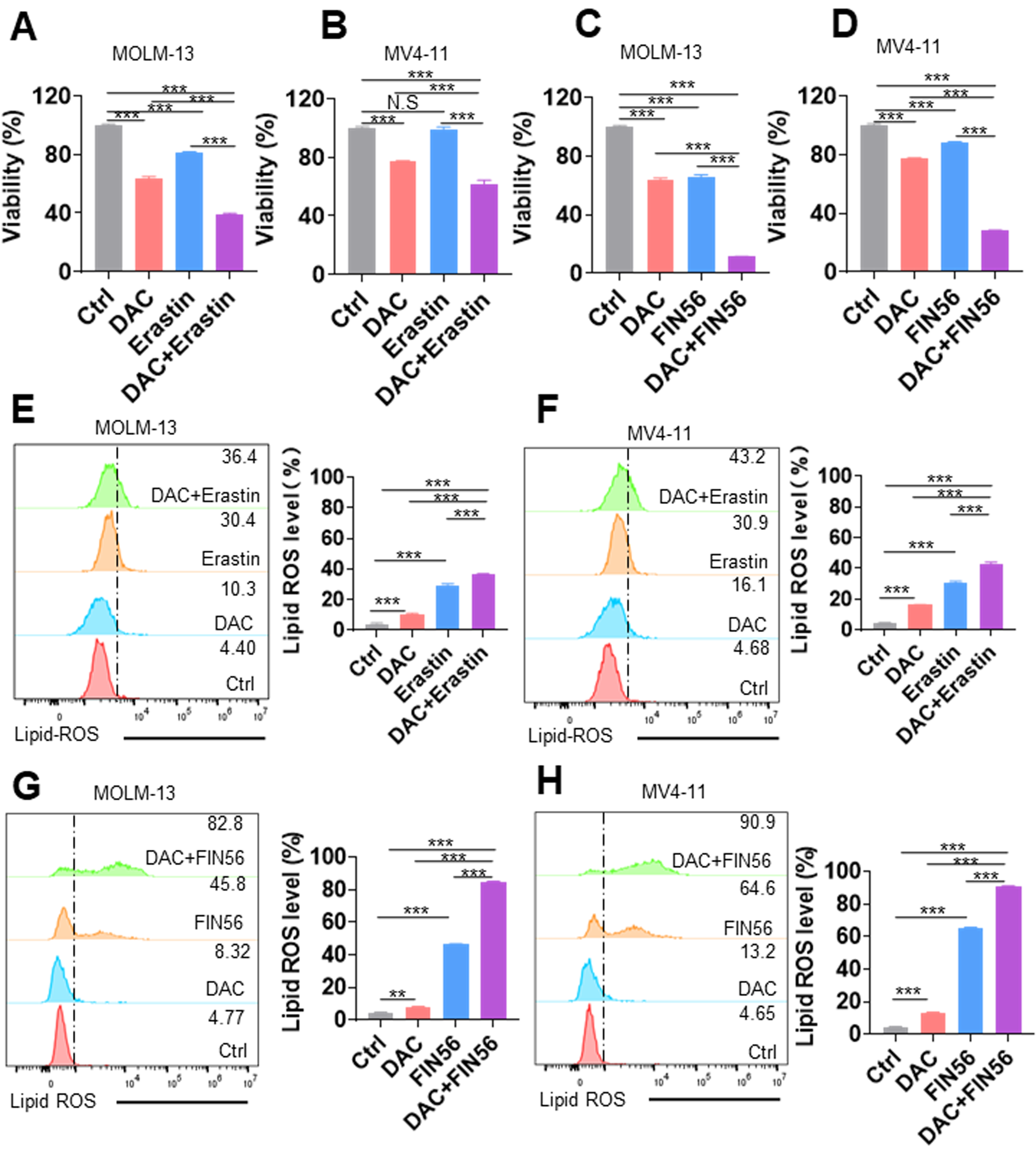

Supplement: Supplementary file 3 — Supplementary Material 3 [file 40164_2024_489_MOESM3_ESM.tif]

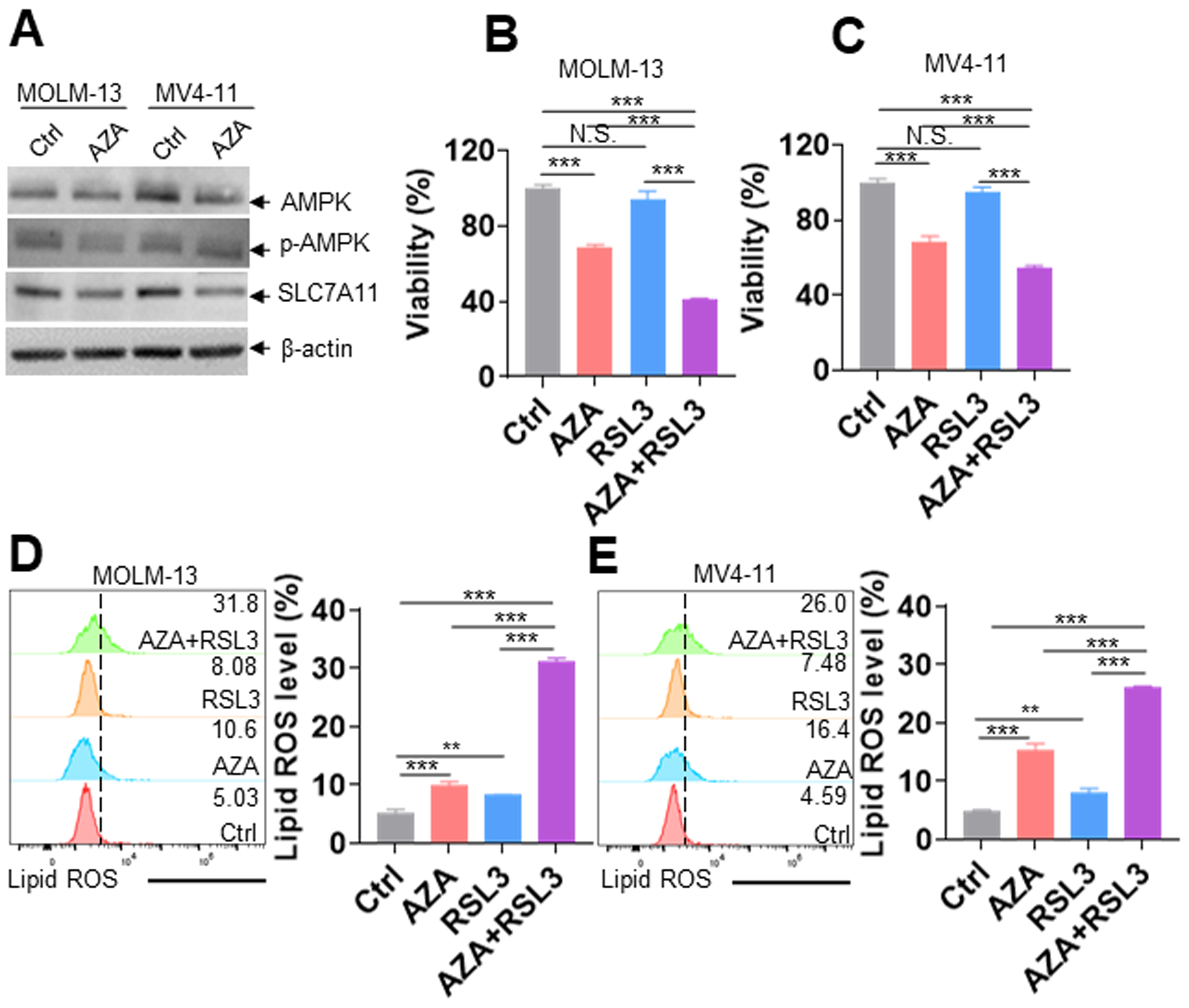

Supplement: Supplementary file 4 — Supplementary Material 4 [file 40164_2024_489_MOESM4_ESM.tif]

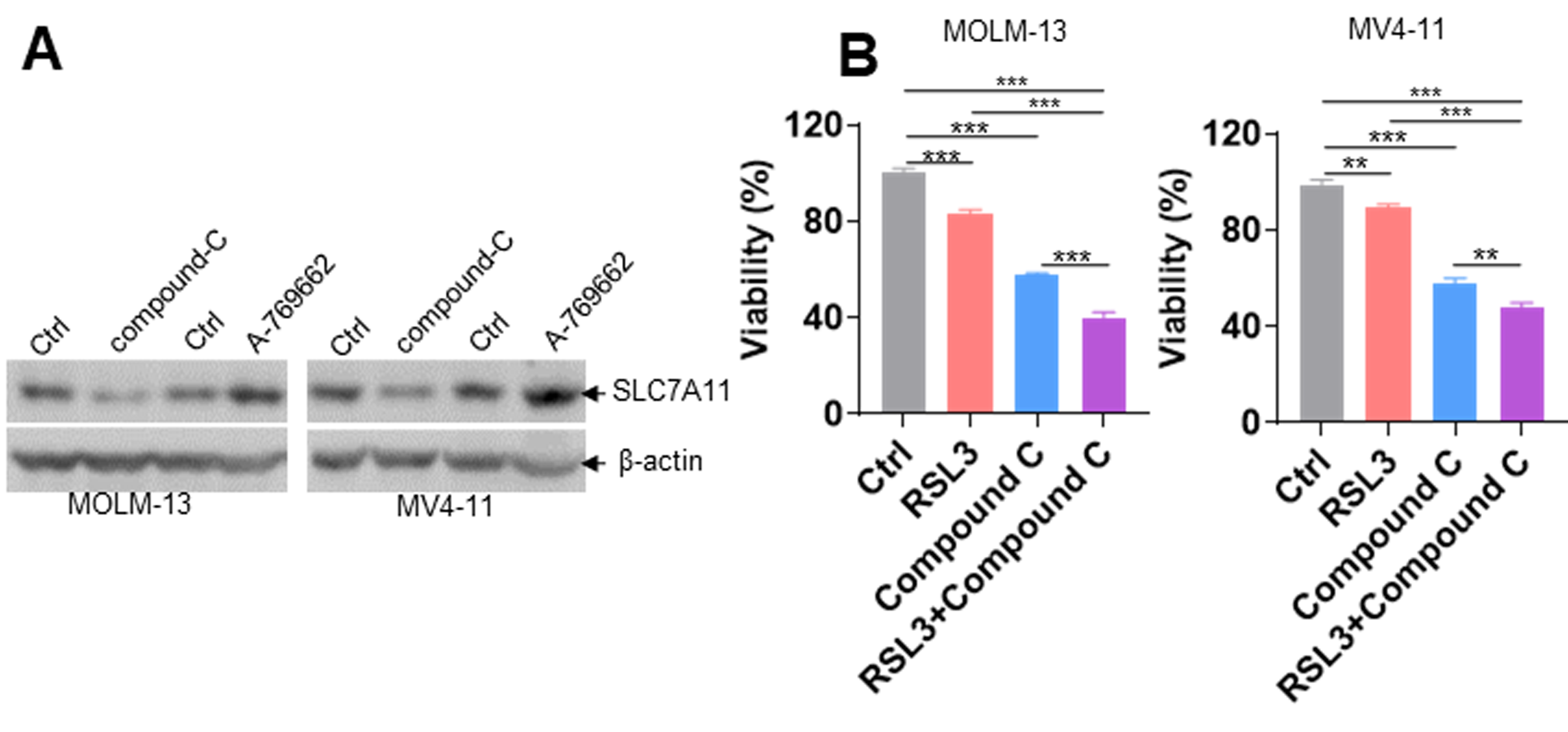

Supplement: Supplementary file 5 — Supplementary Material 5 [file 40164_2024_489_MOESM5_ESM.tif]

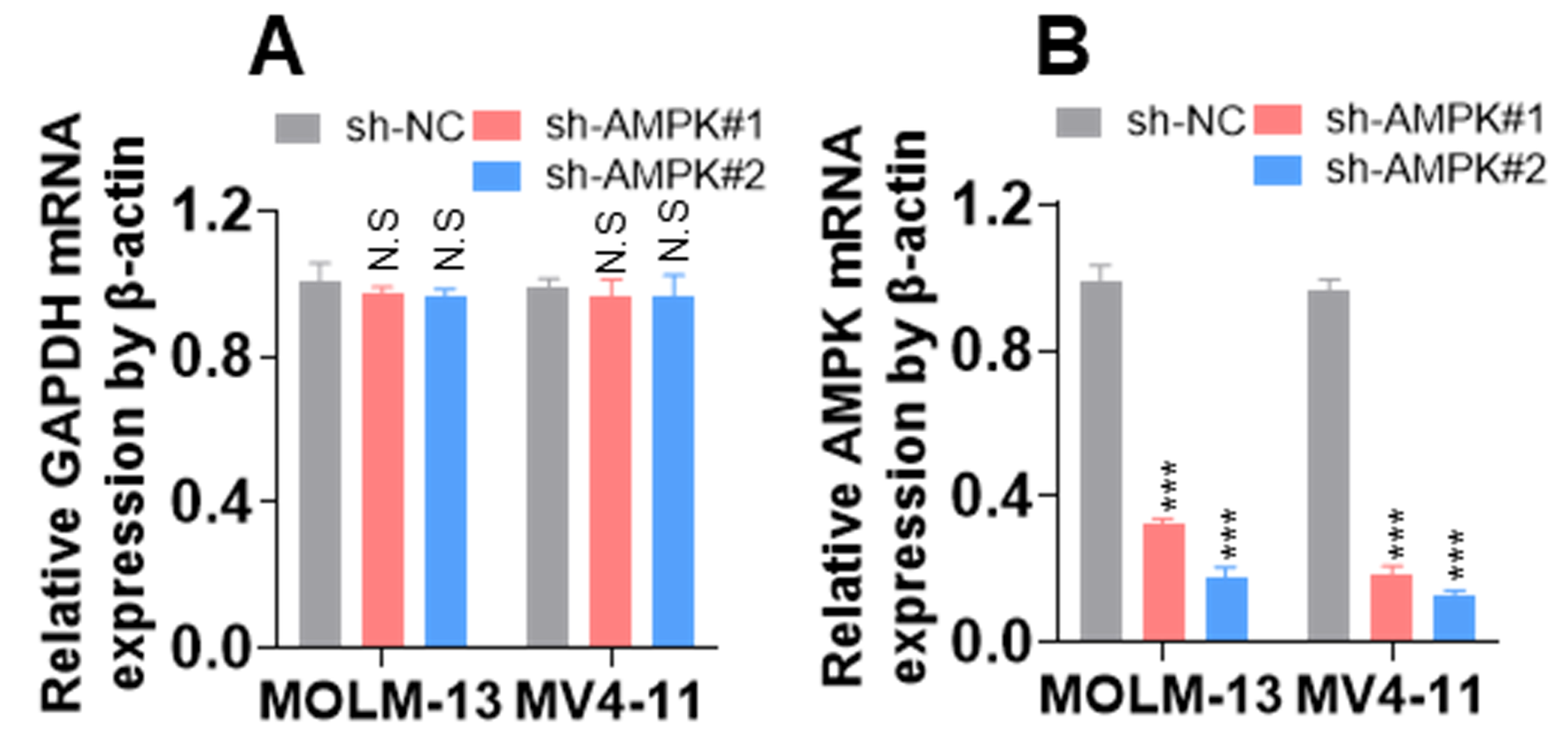

Supplement: Supplementary file 6 — Supplementary Material 6 [file 40164_2024_489_MOESM6_ESM.tif]

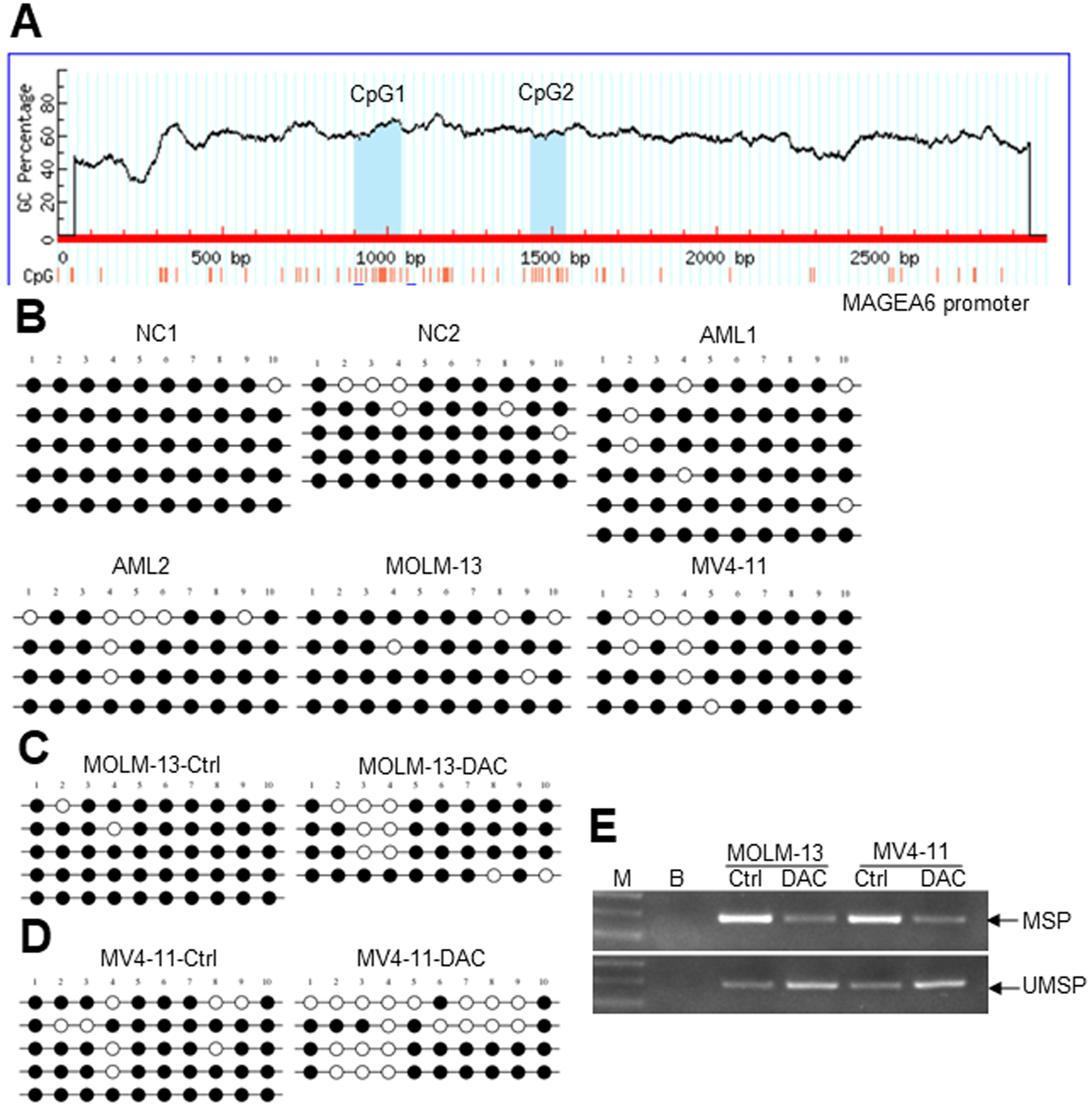

Supplement: Supplementary file 7 — Supplementary Material 7 [file 40164_2024_489_MOESM7_ESM.tif]

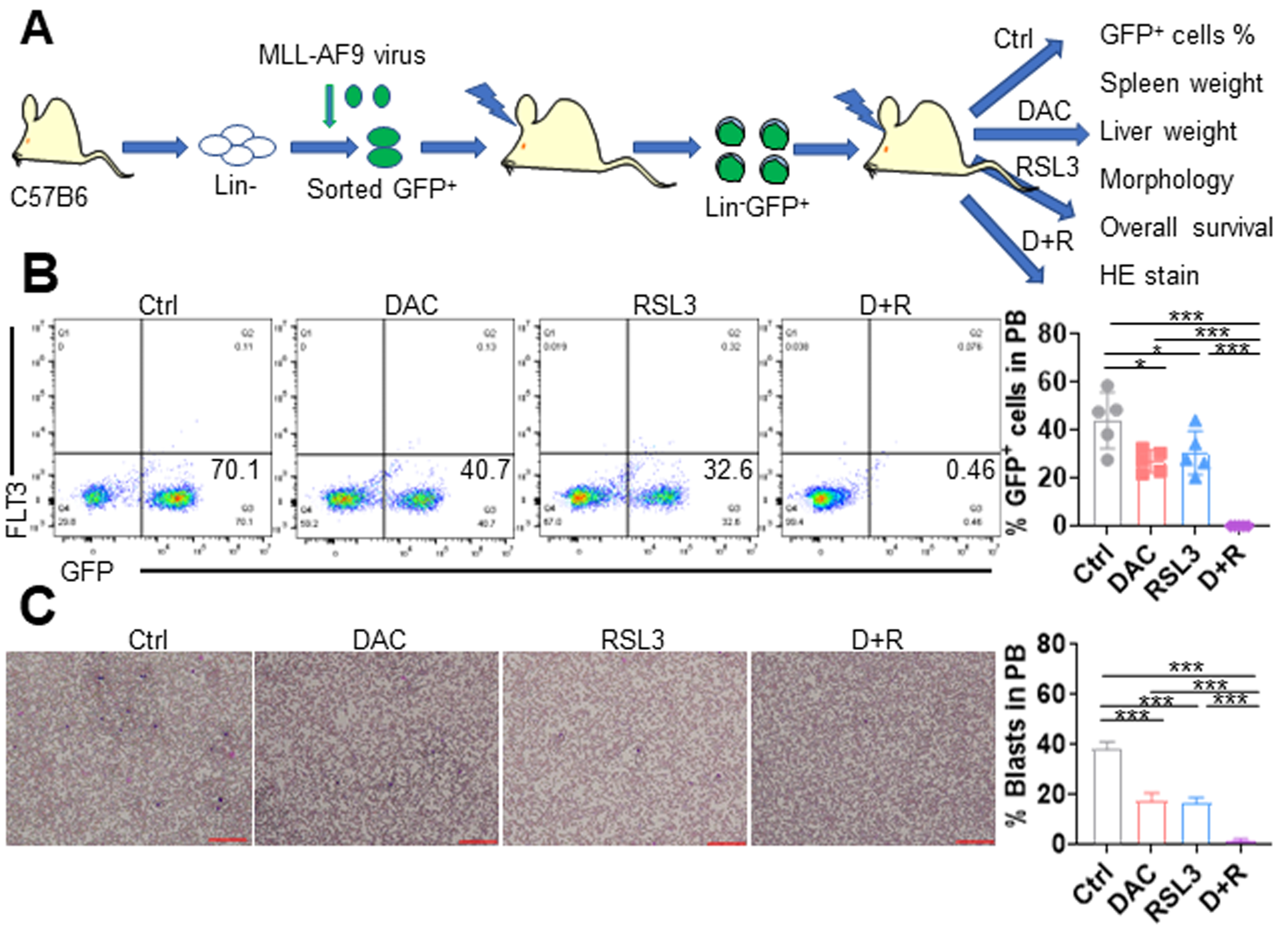

Supplement: Supplementary file 8 — Supplementary Material 8 [file 40164_2024_489_MOESM8_ESM.tif]

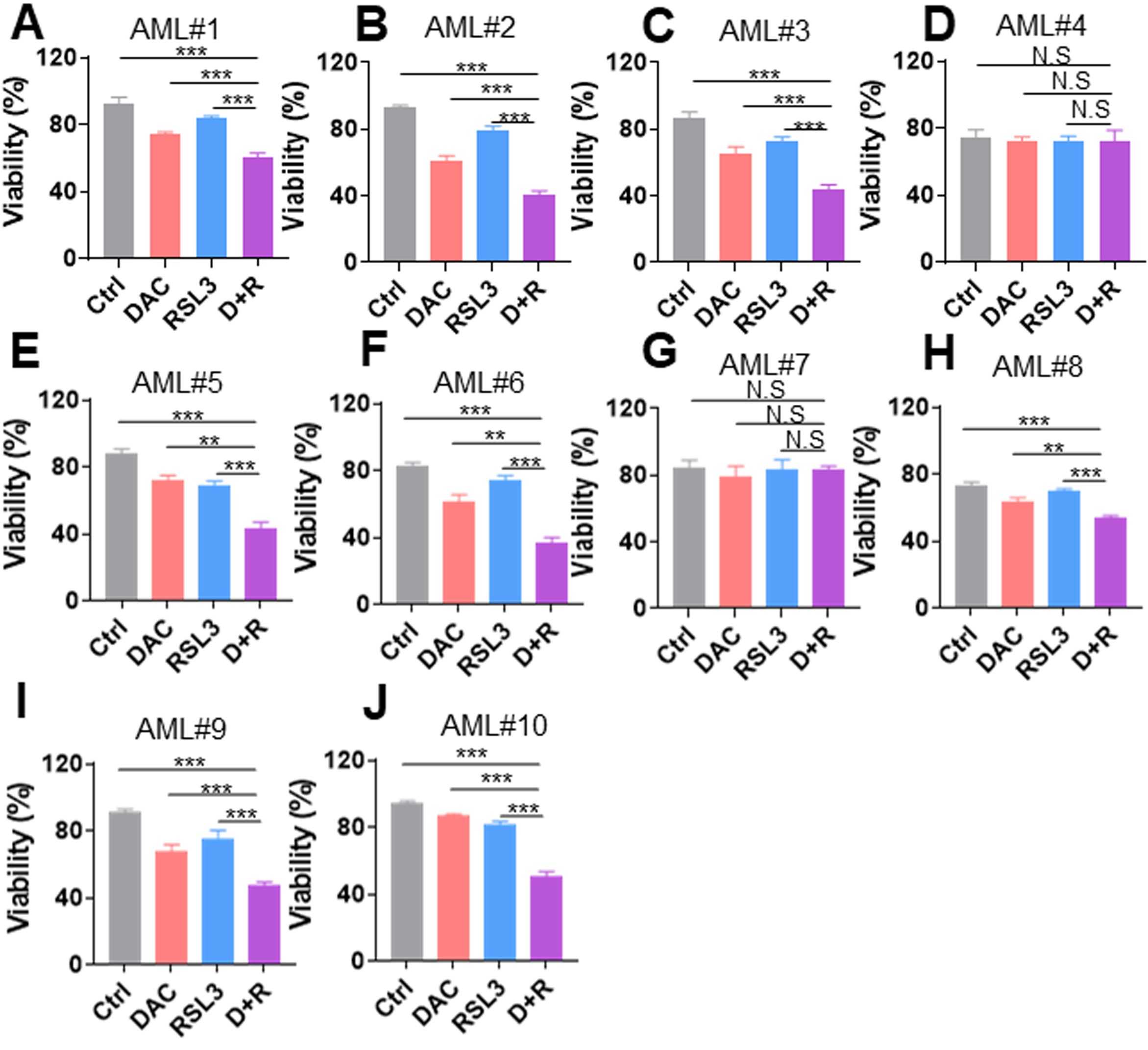

Supplement: Supplementary file 9 — Supplementary Material 9 [file 40164_2024_489_MOESM9_ESM.tif]
