# Supplementary material for: Low-dose hypomethylating agents cooperate with ferroptosis inducers to enhance ferroptosis by regulating the DNA methylation-mediated MAGEA6-AMPK-SLC7A11-GPX4 signaling pathway in acute myeloid leukemia
Source: Exp Hematol Oncol. 2024 Feb 20;13:19. doi: 10.1186/s40164-024-00489-4 (PMC10877917; doi:10.1186/s40164-024-00489-4)
Supplement: Supplementary file 10 — Supplementary Material 10 [file 40164_2024_489_MOESM10_ESM.docx]

Table S1: Detailed characteristics of AML patients

| N | Gender | Age (year) | Diagnosis | FAB type | Blasts% | Cytogenetics | Molecular |
| --- | --- | --- | --- | --- | --- | --- | --- |
| 1 | F | 53 | De novo | M1 | 83 | 46, XX | FLT3-TID, NPM1 |
| 2 | F | 59 | De novo | M2b | 91 | 46, XX, t(8;21)  (q22;q22) | AML1-ETO |
| 3 | M | 50 | Refractory | M5 | 95 | 46, XY | CEBPα |
| 4 | F | 61 | De novo | M5 | 89 | Complex karyotype | FLT3-ITD |
| 5 | M | 45 | De novo | M5 | 75 | 46, XY | CEBPα |
| 6 | F | 52 | Refractory | M4 | 78 | 46, XX | WT1 |
| 7 | M | 58 | De novo | M2 | 82 | 45, XY, -7, add(8)(p11.2) | WT1 |
| 8 | M | 37 | De novo | M4 | 93 | 46,XY,der(9)t(9;11) (p22:q23) | MLL-AF9 |
| 9 | M | 48 | De novo | M5 | 83 | 46, XY, t(9;11) (p22:q23) | MLL-AF9 |
| 10 | F | 56 | Refractory | M4 | 76 | 46, XX | CEBPα |
