# Supplementary material for: Low-dose hypomethylating agents cooperate with ferroptosis inducers to enhance ferroptosis by regulating the DNA methylation-mediated MAGEA6-AMPK-SLC7A11-GPX4 signaling pathway in acute myeloid leukemia
Source: Exp Hematol Oncol. 2024 Feb 20;13:19. doi: 10.1186/s40164-024-00489-4 (PMC10877917; doi:10.1186/s40164-024-00489-4)
Supplement: Supplementary file 11 — Supplementary Material 11 [file 40164_2024_489_MOESM11_ESM.docx]

**Table S2: The sequences of primers for qRT-PCR and construction of plasmids**

| Genes | Sequences |
| --- | --- |
| PTGS2-L | 5′-CGG TGA AAC TCT GGC TAG ACA G-3′ |
| PTGS2-R | 5′-GCA AAC CGT AGA TGC TCA GGG A-3′ |
| GPX4-L | 5′-GCA ACC AGT TTG GGA GGC AGG AG-3′ |
| GPX4-R | 5′-CCT CCA TGG GAC CAT AGC GCT TC-3′ |
| AMPK-L | 5′-TTG AAA CCT GAA AAT GTC CTG CT-3′ |
| AMPK-R | 5′-GGT GAG CCA CAA CTT GTT CTT-3′ |
| SLC7A11-L | 5′-TCT CCA AAG GAG GTT ACC TGC-3′ |
| SLC7A11-R | 5′-AGA CTC CCC TCA GTA AAG TGA C-3′ |
| MAGEA3-L | 5′-CAG AGG AGT CAG CAC TGC AA-3′ |
| MAGEA3-R | 5′-GAC TCT GGG GAG GAT CTG GT-3′ |
| MAGEA6-L | 5′-ACC AGA TCC TCC CCA GAG TC-3′ |
| MAGEA6-R | 5′-TGA ACC AAC TTG GC CAC CTT-3′ |
| GAPDH-L | 5′-CCG GGA AAC TGT GGC GTG ATG G-3′ |
| GAPDH-R | 5′-AGG TGG AGG AGT GGG TGT CGC TGT T-3′ |
| β-actin-L | 5′-CTC TTC CAG CCT TCC TTC CT-3′ |
| β-actin-R | 5′-AGC ACT GTG TTG GCG TAC AG-3′ |
| *MSP-MAGEA6-L | 5′-TAG TAT CGT TGT TAG GAT GTG ACG T-3′ |
| *MSP-MAGEA6-R | 5′-AAC CCT CTA TCT AAA ATA AAA CCC G-3′ |
| *UMSP-MAGEA6-L | 5′-TAG TAT TGT TGT TAG GAT GTG ATG T-3′ |
| *UMSP-MAGEA6-R | 5′-AAC CCT CTA TCT AAA ATA AAA CCC AC-3′ |
| ^#^Bis-MAGEA6-L | 5′-GGG TTT TGT TTT TGG TAT TAA GTT A-3′ |
| ^#^Bis-MAGEA6-R | 5′-AAC ATA CTA AAT CCC CTC AAA ACTC-3′ |
| ^&^sh-AMPK#1 | 5′-GTT GCC TAC CAT CTC ATA ATA-3′ |
| ^&^sh-AMPK#2 | 5′-CCT GGA AGT CAC ACA ATA GAA-3′ |
| ^&^sh-GPX4#1 | 5′-GTG AGG CAA GAC CGA AGT AAA-3′ |
| ^&^sh-GPX4#2 | 5′-GTG GAT GAA GAT CCA ACC CAA-3′ |
| ^&^OE-MAGEA6-L | 5′-GGA TCTA TTT CCG GTG AAT TCA TGC CTC TTG AGC AGA GGA GTC-3′ |
| ^&^OE-MAGEA6-R | 5′-GGA GGG AGA GGG GCG GGA TCC TCA CTC TTC CCC CTC TCT CAA A-3′ |
| ^&^OE-SLC7A11-L | 5′-GGA TCT ATT TCC GGT GAA TTC ATG GTC AGA AAG CCT GTT GTG TC-3′ |
| ^&^OE-SLC7A11-R | 5′-GGG ATC CGC GGC CGC TCT AGA TCA TAA CTT ATC TTC TTC TGG TAC AAC TTC-3′ |

*for methylation-specific PCR (MSP) and unmethylation-specific PCR (UMSP).

^#^for bisulfite (Bis)-sequencing analysis.

^&^for construction of plasmids.
