# Supplementary material for: Low-dose hypomethylating agents cooperate with ferroptosis inducers to enhance ferroptosis by regulating the DNA methylation-mediated MAGEA6-AMPK-SLC7A11-GPX4 signaling pathway in acute myeloid leukemia
Source: Exp Hematol Oncol. 2024 Feb 20;13:19. doi: 10.1186/s40164-024-00489-4 (PMC10877917; doi:10.1186/s40164-024-00489-4)
Supplement: Supplementary file 12 — Supplementary Material 12 [file 40164_2024_489_MOESM12_ESM.docx]

**Supplemental material and methods**

**Chemical Regents**

Carbobenzoxy-valyl-alanyl-aspartyl-[O-methyl]-fluoromethylketone (Z-VAD-FMK, Beyotime Biotechnology, Nanjing, Jiangsu, China), Ac-DEVD-CHO (Ac-DEVD, Beyotime Biotechnology, China), 5-Azacitidine (AZA, Sigma-Aldrich, St. Louis, MO, USA), 5-aza-2'-deoxycytidine (Decitabine, DAC, Sigma-Aldrich), (1S,3R)-RSL3 (TargetMol, Boston, MA, USA), erastin (TargetMol), compound C (TargetMol), A-769662 (TargetMol), Ferrostatin-1 (Fer-1, TargetMol),

chloroquine (CQ, TargetMol), necrostatin-1 (Nec-1, TargetMol), FIN56 (TargetMol), 5-fluorouracil (Sigma-Aldrich), and puromycin (puro, Sigma-Aldrich) were dissolved in dimethyl sulphoxide and kept at -20 ℃ until use.

**Western blot**

Western blot analysis was performed by standard techniques. Briefly, all leukemic cells were collected and lysed by 1×RIPA lysis buffer with phenylmethylsulfonyl fluoride, sodium orthovanadate, phosphatase, and protease inhibitors (Beyotime Biotechnology). Extracted protein was heated at 100 ℃ for 5 min and rapid cooling at 4 ℃ for 5 min. Then, all protein concentrations were measured by a bicinchoninic acid assay (BCA, Thermo Scientific). Proteins (20 μg/well) were fractionated by electrophoresis through polyacrylamide gels (Bio-Rad, Richmond, CA, USA) and transferred to a PVDF membrane (0.45 μm). After incubation with TBS with 5% skim milk powder for 2 h at room temperature, blots were incubated with primary antibodies overnight at 4 °C. Blots were washed, and signals were measured by chemiluminescence reagents (Bio-Rad) with an imaging system (Bio-Rad), after incubated with a secondary HRP-conjugated antibody for 1 h at room temperature. The following antibodies were used: AMPK (#5831, Cell Signaling Technology, Beverly, MA, USA), p-AMPK (Ser485, #2537, Cell Signaling Technology), SLC7A11 (26864-1-AP, Proteintech Group, Inc Rosemont, IL, USA), GPX4 (67763-1-Ig, Proteintech Group), MAGEA6 (14602-1-AP, Proteintech Group). As necessary, blots were stripped and reprobed with β-actin antibody (ab6276, Abcam) as an endogenous control.

**CCK-8 assay**

All leukemic cells (100 μl, 1×10^5^ cells/mL) treated with or without compounds were seeded in 96-well plates. After plating for 24 h, CCK-8 solution (10 μL, TargetMol) was added in and incubated for 4 h at 37 °C incubator. The absorbance was measured at 450 nm by an MRX II microplate reader (Dynex, Chantilly, VA, USA). Cell viability was calculated by CCK-8 assay or trypan blue staining (Thermo Fisher Scientific).

**Lipid reactive oxygen species (ROS) level assay**

Lipid ROS was detected by C11-BODIPY® 581/591 (D3861, Thermo Fisher Scientific) staining. C11-BODIPY was incubated in a complete RPMI-1640 medium containing leukemic cells at 37 °C for 30 min. The intensity of lipid-ROS-FITC was measured by flow cytometry at 488 nm.

**Malondialdehyde (MDA) assay**

MDA amounts (nmol/mgprot) were measured by a Lipid Peroxidation MDA Assay Kit (A003-4-1, Nanjing Jiancheng Bioengineering Institute, Nanjing, Jiangsu Province, China) according to the manufacturer’s instructions. Briefly, treated or untreated leukemic cells were collected and lysed by Ultrasonication. [Liquid supernatant](javascript:;)s were mixed with thiobarbituric acid, and the OD value was measured at 532 nm by an MRX II microplate reader (Dynex).

**Glutathione (GSH) assay**

We used a whole glutathione assay kit (A006-2, Nanjing Jiancheng Bioengineering Institute, China) with enzyme labeling to analyze GSH amounts following the manufacturer's instructions. Briefly, AML cells were harvested and lysed in 1×PBS by ultrasonic pyrolysis. The supernatant was mixed equally with the precipitant agent and centrifuged at 3500×rpm for 10 min. Finally, the OD value was measured at 405 nm by an MRX II microplate reader (Dynex).

**GPX4 enzyme activity analysis**

GPX4 inhibitor screening assay kit (#701880, Cayman Chemicals, Ann Arbor, Michigan, USA) was used to measure GPX4 enzyme activity according to manufacturer's instructions. Briefly, GPX4 assay buffer and diluted GPX4 enzyme buffer were mixed and added in 96-well plate. Positive control GPX4 inhibitor (ML-162) was added in wells for 100% inhibitor control. Treated and untreated AML cells were collected and lysed in GPX4 sample buffer containing 1 mM DTT, 5 mM EDTA (pH7.5), 50 mM Tris-HCl, followed by incubation at room temperature for 60 min and plated in 96-well plate. Glutathione and glutathione reductase were mixed and added in all wells, followed by the reconstituted NAPDH and cumene hydroperoxide. Finally, cumene hydroperoxide was added in all wells and absorbance was measured at 340 nm using an MRX II microplate reader (Dynex) every 60 seconds for 5 minutes. The rate of decrease in OD 340 is directly proportional to GPX4 enzyme activity.

**RNA sequencing (RNA-seq) analysis**

MOLM-13 cells treated with or without DAC (0.5 μM) for 48 h, and total RNA was extracted by Trizol reagent (Invitrogen, USA) according to the manufacturer's protocol. Then, mRNA enrichment, fragmentation, and cDNA synthesis were performed to construct cDNA library using KAPA Stranded RNA-Seq Library Preparation Kit (Illumina Technologies, San Diego, CA, USA). Illumina HiSeq 4000 sequencing platform was used to perform sequencing. Illumina/Solexa Pipeline Image analysis as used to assess base calling and error estimation. Two Software including HISAT2 and StringTie, were committed to constructing the transcriptome and mapping to the corresponding reference genome. After the significant analysis, the DESeq algorithm was applied to filter the differentially expressed genes. Fold change (>2 or < 0.5) and FDR (<0.05) analysis were considered as the criteria for differentially expressed genes (NovelBio Co., Ltd, Shanghai, China).

**Intracellular iron assay**

Intracellular iron levels were measured by FerroOrange (Dojindo, Japan). AML cells (2×10^5^/ml) were seeded in 12-well plates, and FerroOrange was added (final concentration, 1 mM) in the medium for the incubation of 30 min at 37 °C. The fluorescence was measured by flow cytometry at 488 nm.

**Engraftment of NOD/SCID‑IL2Rγ mice (NSG)**

Female NSG mice (8-week-old) were intraperitoneally injected by busulfan (30 mg/kg, Sigma) (Shanghai Model Organisms Center, Shanghai, China) one day before transplantation. Every NSG mouse was xenografted with 1×10^6^ R/R AML blasts by vein injection. Mice were randomly divided into four groups: Ctrl, DAC (2.5 mg/kg), RSL3 (5 mg/kg), DAC (2.5 mg/kg) +RSL3 (5 mg/kg). RSL3 and DAC were dissolved in solution with 10% DMSO + 45% PEG300 + 45% Saline. The control mice received the placebo (10% DMSO + 45% PEG300 + 45% Saline). Treatment (three times per week for total two weeks) was started at three weeks after transplantation. Survival time was determined from the first day of transplantation until death.
