# Supplementary material for: Low-dose hypomethylating agents cooperate with ferroptosis inducers to enhance ferroptosis by regulating the DNA methylation-mediated MAGEA6-AMPK-SLC7A11-GPX4 signaling pathway in acute myeloid leukemia
Source: Exp Hematol Oncol. 2024 Feb 20;13:19. doi: 10.1186/s40164-024-00489-4 (PMC10877917; doi:10.1186/s40164-024-00489-4)
Supplement: Supplementary file 13 — Supplementary Material 13 [file 40164_2024_489_MOESM13_ESM.docx]

Supplemental Figure legend

**Fig S1. DAC cooperates with RSL3 to enhance anti-leukemogenesis in AML cells.** (A and B) Cell viability was measured in MOLM-13 and MV4-11 cells, which were treated with Ctrl, indicated concentrations of DAC for 48 h, indicated concentrations of RSL3 for 24 h, or their combination. CalcuSyn was used to assess the possible synergistic effects of DAC and RSL3 in MOLM-13 and MV4-11 cells. CI < 1.0 is considered a synergistic effect. (C and D) Cell viability was measured in MOLM-13 and MV4-11 cells treated with Ctrl, DAC (0.5 μM) for 48 h, RSL3 (0.05 μM) for 24 h, or their combination. ****P* < 0.001; N.S: not significant.

**Fig S2. Ferroptosis inhibitor Fer-1 rescues the decreased viability induced by DAC+RSL3 cotreatment.** (A and B) Viability was measured in MOLM-13 and MV4-11 cells preincubated with Fer-1 (2 µM), Z-VAD (20 µM), CQ (10 µM), or Nec-1 (50 µM) for 1 h and then treated with Ctrl, DAC (0.5 μM), RSL3 (0.05 μM), or DAC (0.5 μM)+RSL3 (0.05 μM). ****P* < 0.001. N.S: not significant compared with DAC+RSL3 cotreatment (line 4).

**Fig S3. DAC synergizes with Erastin or FIN56 to facilitate ferroptosis in AML cells.** (A and B) Cell viability was measured in MOLM-13 and MV4-11 cells treated with Ctrl, DAC (0.5 μM) for 48 h, Erastin (10 μM) for 24 h, or their combination. (C and D) Cell viability was measured in MOLM-13 and MV4-11 cells treated with Ctrl, DAC (0.5 μM) for 48 h, FIN56 (5 μM) for 24 h, or their combination. (E and F) Lipid ROS levels were measured in MOLM-13 and MV4-11 treated with Ctrl, DAC (0.5 μM) for 48 h, Erastin (10 μM) for 24 h, or their combination. The representative plots (left) and statistical analysis of lipid ROS levels (right) are shown. (G and H) MOLM-13 and MV4-11 cells were incubated with Ctrl, DAC (0.5 μM) for 48 h, FIN56 (5 μM) for 24 h, or their combination, followed by measuring lipid ROS level. The representative plots (left) and statistical analysis of lipid ROS levels (right) are shown. ***P* < 0.01; ****P* < 0.001; N.S: not significant.

**Fig S4. Synergistic activity with AZA and RSL3 by inducing ferroptosis in AML cells.** (A) AMPK, p-AMPK, and SLC7A11 protein levels were measured in MOLM-13 and MV4-11 cells treated with AZA (0.5 μM) for 48 h. (B and C) Cell viability was measured in MOLM-13 and MV4-11 cells treated with Ctrl, AZA (0.5 μM) for 48 h, RSL3 (0.05 μM) for 24 h, or their combination. (D and E) Lipid ROS levels were measured in MOLM-13 and MV4-11 cells incubated with Ctrl, AZA (0.5 μM), RSL3 (0.05 μM), or AZA (0.5 μM)+RSL3 (0.05 μM). The representative plots (left) and statistical analysis of lipid ROS levels (right) are shown. ***P* < 0.01; ****P* < 0.001; N.S: not significant.

**Fig S5. AMPK inhibitor enhances RSL3-induced anti-leukemogenesis ability.** (A) SLC7A11 protein expression was measured in MOLM-13 and MV4-11 cells incubated with Ctrl, AMPK inhibitor Compound C (0.01 μM), or activator A-769662 (50 μM) for 24 h. (B) Viability was measured in MOLM-13 and MV4-11 cells incubated with Ctrl, Compound C (0.01 μM), RSL3 (0.05 μM), or Compound C (0.01 μM)+RSL3 (0.05 μM) for 24 h. ***P* < 0.01; ****P* < 0.001.

**Fig S6.** GAPDH is an appropriate loading control for qRT-PCR. (A) GAPDH mRNA expression was measured in MOLM-13 and MV4-11 cells transduced with shRNAs for AMPK (sh-AMPK#1 and #2) or negative control (sh-NC). *β-actin* was used as a loading control for qRT-PCR. (B) AMPK mRNA expression was measured in MOLM-13 and MV4-11 cells transduced with sh-AMPK or sh-NC using *β-actin* as a loading control for qRT-PCR. ****P* < 0.001; N.S: not significant.

**Fig S7. Hypermethylation at the *MAGEA6* promoter in AML cells.** (A) MethPrimer software was used to determine possible CpG islands at the *MAGEA6* promoter. Two CpG islands were found at the *MAGEA6* promoter. (B) Bisulfite-genomic sequencing was performed in two NCs, two AML samples, and two AML cell lines. Four to six colonies were shown for each sample. Each row of circle represents the sequence of an individual clone. Black and empty circles represent methylated and unmethylated CpG dinucleotides, respectively. (C and D) Bisulfite-genomic sequencing was performed in Ctrl or DAC-treated MOLM-13 (C) and MV4-11 cells (D). (E) Methylation-specific PCR (MSP) and unmethylation-specific PCR (UMSP) were performed to assess the methylation level of CpG island 1 in MOLM-13 and MV4-11 cells treated with or without DAC (0.5 μM) for 48 h. M: DNA Marker; B: Blank.

**Fig S8. DAC and RSL3 have synergistic anti-leukemic activity in the MLL-AF9-transformed murine AML model.** (A) Outline for MLL-AF9-induced murine AML model treated with Ctrl, DAC, RSL3, or DAC+RSL3 (D+R). (B) The frequencies of GFP^+^ cells were measured in blood mononuclear cells from Ctrl, DAC, RSL3, or D+R-treated AML mice. Representative plots (left) and statistical analysis of GFP^+^ cells (right) are shown. (C) The percentage of murine AML blasts was calculated by Wright-Giemsa stain in blood from Ctrl, DAC, RSL3, or D+R-treated AML mice. Shown are the representative pictures (left) and statistical analysis of percentage of blood blasts (right). Bar scales represent 100 μm. **P* < 0.05; ****P* < 0.001.

**Fig S9.** **DAC and RSL3 synergistically have antileukemic effects in primary AML samples.** (A–J) Cell viability was measured in 10 AML samples treated with Ctrl, DAC (1.0 μM) for 48 h, RSL3 (0.1 μM) for 24 h, or their combination. ***P* < 0.01; ****P* < 0.001; N.S: not significant.
